# A cyclin D1 intrinsically disordered domain accesses modified histone motifs to govern gene transcription

**DOI:** 10.1038/s41389-023-00502-1

**Published:** 2024-01-08

**Authors:** Xuanmao Jiao, Gabriele Di Sante, Mathew C. Casimiro, Agnes Tantos, Anthony W. Ashton, Zhiping Li, Yen Quach, Dharmendra Bhargava, Agnese Di Rocco, Claudia Pupo, Marco Crosariol, Tamas Lazar, Peter Tompa, Chenguang Wang, Zuoren Yu, Zhao Zhang, Kawthar Aldaaysi, Ratna Vadlamudi, Monica Mann, Emmanuel Skordalakes, Andrew Kossenkov, Yanming Du, Richard G. Pestell

**Affiliations:** 1 Baruch S. Blumberg Institute, Doylestown, PA 18902 USA; 2 Xavier University School of Medicine at Aruba, Oranjestad, Aruba; 3 Department of Science and Mathematics, Abraham Baldwin Agricultural College, Tifton, GA 31794 USA; 4 Institute of Enzymology, Hun-Ren Research Centre for Natural Sciences, Budapest, Hungary; 5 Division of Cardiovascular Medicine, Lankenau Institute for Medical Research, Wynnewood, PA 19003 USA; 6 Department of Cancer Biology, Thomas Jefferson University, Philadelphia, PA 19107 USA; 7 VIB-VUB Center for Structural Biology, Vrije Universiteit Brussel, Brussels, 1050 Belgium; 8 Research Center for Translational Medicine, Shanghai East Hospital, Tongji University School of Medicine, Shanghai, 200120 China; 9 Department of Obstetrics and Gynecology, University of Texas Health Sciences Center, San Antonio, TX 78229 USA; 10 The Wistar Institute, Philadelphia, PA 19107 USA

**Keywords:** Checkpoint signalling, Breast cancer

## Abstract

The essential G_1_-cyclin, *CCND1*, is frequently overexpressed in cancer, contributing to tumorigenesis by driving cell-cycle progression. D-type cyclins are rate-limiting regulators of G_1_-S progression in mammalian cells via their ability to bind and activate CDK4 and CDK6. In addition, cyclin D1 conveys kinase-independent transcriptional functions of cyclin D1. Here we report that cyclin D1 associates with H2B^S14^ via an intrinsically disordered domain (IDD). The same region of cyclin D1 was necessary for the induction of aneuploidy, induction of the DNA damage response, cyclin D1-mediated recruitment into chromatin, and CIN gene transcription. In response to DNA damage H2B^S14^ phosphorylation occurs, resulting in co-localization with γH2AX in DNA damage foci. Cyclin D1 ChIP seq and γH2AX ChIP seq revealed ~14% overlap. As the cyclin D1 IDD functioned independently of the CDK activity to drive CIN, the IDD domain may provide a rationale new target to complement CDK-extinction strategies.

## Introduction

Genome instability is a hallmark of cancer cells [1]. Chromosome instability (CIN) is often mutually exclusive from hypermutation genotypes and represents a distinct subtype of genome instability. CIN is an early event in tumor formation that causes either the inactivation of tumor suppressor genes, through defects in genome maintenance and destabilization of the nucleotide sequence or accelerates the gain of oncogenic loci. Aneuploidy, also known as somatic copy number alterations (SCNAs), is widespread in human cancers [2–6]. For most tumors, there is a positive correlation between SCNA levels and the total number of mutations. Although CIN is likely due to defects in a network of genes that regulate mitotic checkpoints, collaborative oncogenes, including c-Myc [7] or cyclin D1 [8–10] are sufficient for the induction of CIN. The induction of cellular growth and CIN by c-Myc involves dissociable domains [7], however, the domain of cyclin D1 required for the induction of CIN is unknown.

*Cyclin D1* overexpression occurs in human breast, prostate, lung, and gastrointestinal malignancies and its abundance is induced at the level of transcription, translation and post-translational modifications [11, 12]. Phosphorylation and inactivation of the retinoblastoma (RB), the nuclear respiratory factor 1 (NRF1), and several other proteins by the cyclin D1/CDK4/6 complex is known to govern nuclear DNA synthesis and mitochondrial biogenesis [13–16]. Oncogenic signals induce *cyclin D1* through multiple processes, including transcription, alterations in protein stability and miRNA which bind and regulate cyclin D1 via the 3’-UTR [17, 18]. In murine models, cyclin D1 was required for the growth of ErbB2-induced mammary tumors [19]. The murine *cyclin D1* gene was shown to be required for Ras- or ErbB2-induced mammary tumorigenesis and APC-induced gastrointestinal tumorigenesis in *cyclin D1* gene deletion mice [20, 21]. Although selective inhibitors of cyclin D1/CDK4/6 complexes have transitioned effectively to the clinic improving progression-free survival, therapy resistance subsequently arises [17, 22].

In addition to its well-documented function as the regulatory subunit of an RB kinase, cyclin D1 conducts important non-canonical activities, including the induction of cell migration [23], coordination of gene transcription [12, 14], the induction of chromosomal instability (CIN), and the induction of DNA damage repair (DDR) [24–26]. The DDR is associated with the induction of H2AX (Ser139P) and H2B (Ser14P) phosphorylation to establish a heterochromatin state which concentrates DNA repair factors [27]. Similar to several other proteins participating directly in the DDR [28], cyclin D1 can be recruited into chromatin and augments the DDR [25]. Cyclin D1 also regulates E2F-independent transcription through poorly understood mechanisms involving altering transcription factor (TF) recruitment into chromatin [20], binding in chromatin to induce gene transcription [29, 30], confirmed by genome-wide analysis [8, 31]. Genome-wide ChIP-seq studies identified regions of genes to which cyclin D1 was significantly recruited in chromatin with a preference for CTCF binding sites [8, 32], with similar levels of cyclin D1 enrichment found in other studies [8, 9, 31–33]. In vivo studies established that endogenous cyclin D1 is required for a substantial proportion of androgen-mediated gene expression in the prostate and estrogen-responsive gene expression in the mammary gland [34, 35]. Acute activation of a cyclin D1 cDNA, in either tissue culture or the murine mammary gland, induced gene expression within functional pathways governing, cell-cycle control, mitochondrial biogenesis, and the induction of chromosomal instability (CIN) and CIN gene expression [8]. The recruitment of cyclin D1 into chromatin at target gene promoters has been shown in fibroblasts [8, 9], neural [32] and lymphoma cells [33]. Furthermore, other components of the cell-cycle control apparatus have been identified in chromatin including CDKs and p27/p21 [36].

The molecular mechanisms by which a diverse array of genes may be coordinately regulated by cyclin D1 and the mechanisms by which cyclin D1 is recruited into chromatin to regulate gene transcription is unknown. Cyclin D1 does not contain transcriptional co-regulator domains which recognize histone motifs that have been modified by acetylation, phosphorylation, methylation, or citrullination, PHD (plant homeodomain) fingers and BAH (bromo-adjacent homology) domains, “Royal Family,” ADD (ATRX-DNMT3A-DNMT3L) domains or 14-3-3 histone interaction residues. In addition to reader domains, intrinsically disordered regions (IDR) govern protein-protein interactions. IDRs, constitute a substantial fraction of the eukaryotic proteome [37, 38]. Due to their large accessible surface area and inherent flexibility, IDRs are often involved in molecular recognition and protein-protein interaction events [39]. Intrinsically disordered proteins (IDPs) or regions of proteins (IDRs) abound in proteins with signaling functions and are found in TFs and proteins with chromatin-organizing function [40]. IDRs often mediate protein-protein interactions, undergoing induced folding that uncouples specificity from binding strength [41] and mediates distinct functions [42]. Furthermore, specific protein-protein interaction can occur even without full folding (“fuzzy” interactions [43]), as occurs in liquid-liquid phase separation [44]. Even in these cases, the interaction can carry a significant element of specificity, likely arising in an ensemble-encoded, “emergent” way [45]. IDPs/IDRs are frequently involved in dynamic interactions in signaling networks where fine-tuning of the cascade of interactions is exerted through multisite dependence. The current studies identified the cyclin D1 IDR as required for both DNA damage signaling and transcription, serving as a chromatin interaction interface binding H2B^Ser14P^.

## Results

### The cyclin D1 carboxyl terminal region is required to selectively associate with Histone H2B^Ser14P^

**Fig. 1:**
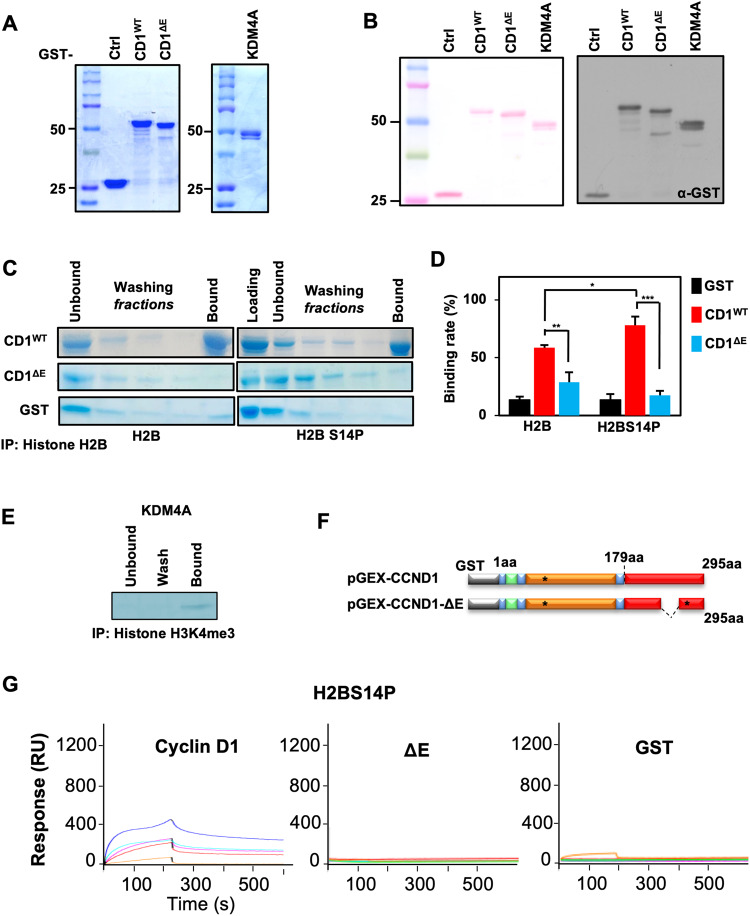
The cyclin D1 carboxyl terminal E-rich region is necessary for the recognition of modified histones by Surface plasmon resonance. **A** Coomassie staining and (**B**). Western blot for cyclin D1 (CD1^WT^), cyclin D1 E-region deleted (CD1^ΔE^) and KDM4A GST fusion proteins. **C** GST pull-down, with (**D**). quantitation shown as mean ± SEM for multiplicate experiments for GST-CD1^WT^, GST-CD1^ΔE^ or GST-ctrl fusion proteins with histone peptide (H2B) and S14-phosphorylated histone peptide (H2BS^14P^). In comparison, GST pull-down of histone H3K4^me3^ with KDM4A is shown in (**E**). **F** Schematic representation of GST-cyclin D1 wild type and ΔE mutant. **G** Surface plasmon resonance (SPR) analysis of histone peptide (H2B^S14P^) with GST-cyclin D1, GST-ΔE or GST-ctrl fusion proteins. (GST-Cyclin D1: blue:10 μM, light blue:7 μM, magenta:5 μM, red:3.5 μM, orange:1 μM).

Prior studies have shown that cyclin D1 is recruited to sites of DNA damage with γH2AX [24–26]. Such sites are known to co-localize with H2B^Ser14P^ [27]. We, therefore, sort to determine whether cyclin D1 is directly associated with H2B or H2B^Ser14^. GST pull-down experiments conducted with cyclin D1 and synthetic modified histone peptides showed cyclin D1 binds H2B^S14P^ with increased affinity compared with GST alone and binds with higher affinity to a peptide encoding H2B^S14P^ (Fig. 1C, D). Although cyclin D1 does not encode known histone protein interaction motifs (PHD fingers, Bromodomain tandem, Chromodomain, Tudor, MET, PWWP, WD40, YEATS, and the β propeller motifs), a glutamate-rich region (poly E region, aa 272-280), which have been identified in IDD, was considered as a candidate motif (Fig. 1A). A GST protein encoding a deletion of the glutamate rich region (cyclin D1ΔE) showed substantially decreased binding to the H2B^S14P^ peptide (Fig. 1C, D). GST control had no signal (Fig. 1C, D). Consistent with prior studies, the GST-KDM4A fusion protein bound to an H3^K4me3^ peptide (Fig. 1E). We further investigated the strength of the interaction between cyclin D1 and modified histones using Surface Plasmon Resonance (SPR). This approach showed a strong and specific binding between cyclin D1 and H2BS14^P^ when compared to GST control with significant binding to the H2B^S14P^ peptide (Fig. 1F, G). The interaction with the H2B^S14P^ peptide was abolished when the cyclin D1 ΔE mutant was introduced (Fig. 1G).

**Fig. 2:**
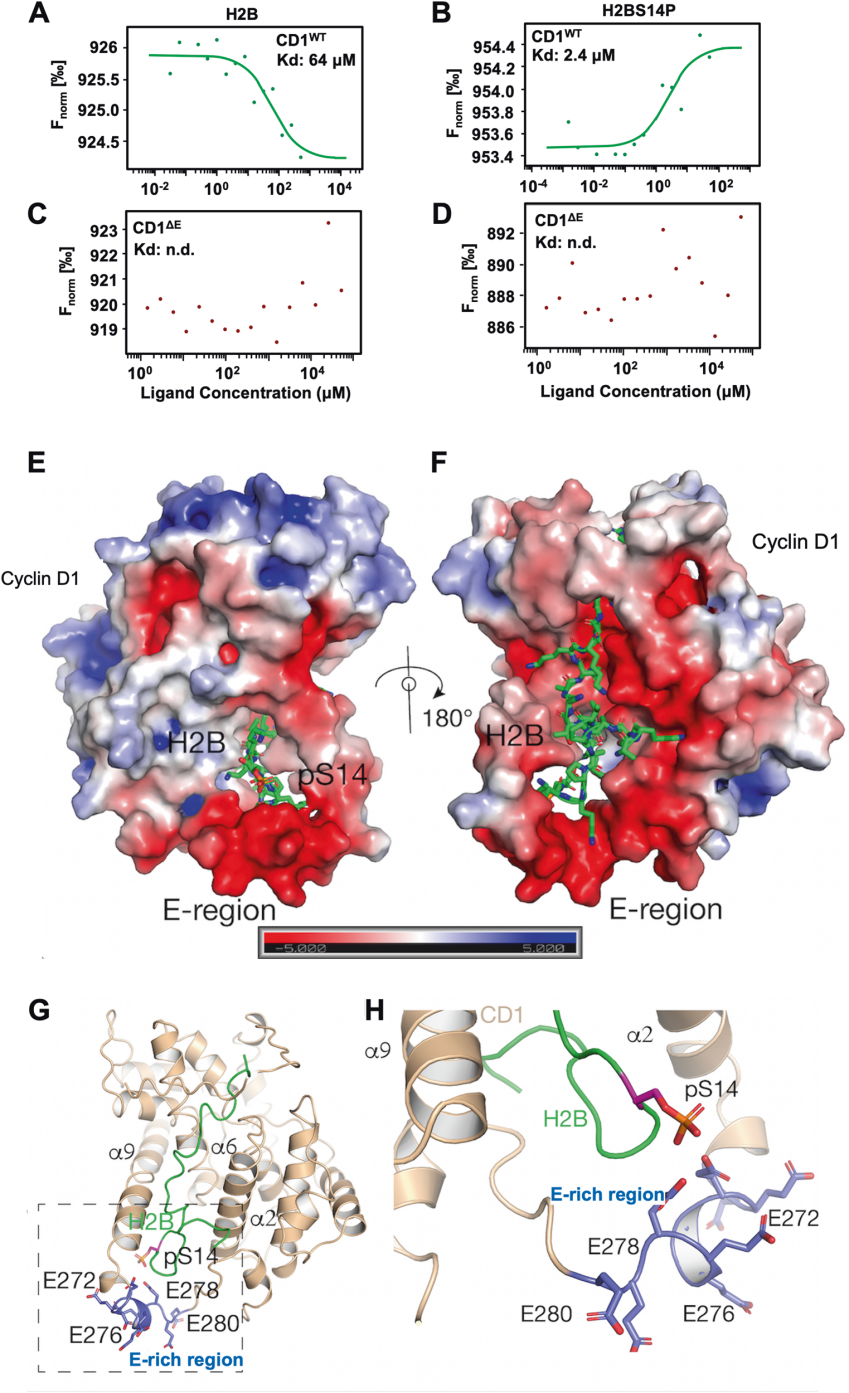
The cyclin D1 E-rich region is necessary for the recognition of H2B^S14P^ in Microscale Thermophoresis (MST) analysis. **A** MST analysis with H2B (**A**, **C**) and H2B^S14P^ (**B**, **D**), indicating Kd, or the lack of binding (Kd cannot be determined, n.d.). **E** Human cyclin D1 (CD1) (Alpha fold – AF-P24385-F1) is shown in electrostatic surface and H2B (PDB ID: 2RVQ) [102] is shown in the sticks (green or magenta). Cyclin D1 contains a large negatively charged cavity on its surface which is ideal for binding to the highly positively charged tail of H2B. It is worth noting that this pocket is formed by helices α2, α 6, and α9 and is away from the CDK4/6 interacting site of cyclin D1. **F** 180° rotation of the structure in (**E**). **G** Cartoon representation of cyclin D1-H2B structural model. Helices α2, α6, and α9 that form the H2B binding pocket of cyclin D1 are shown, as well as the pS14 and the glutamates that form the E-rich region. **H** Zoom in on the representation of the interaction between cyclin D1 and H2B same orientation as in (**H**). Cyclin D1 is shown in wheat color. The E-region which binds S14P is shown in blue. The phosphoserine S14P is shown in magenta on the green sticks of the histone peptide.

We further investigated the nature of the interaction between cyclin D1 and modified histones using microscale thermophoresis (MST). MST showed a strong and specific binding between cyclin D1 and H2B^S14P^ when compared to the binding of unmodified H2B (Kd = 2.4 μM *vs*. 64 μM) (Fig. 2A, B). The interaction with both H2B and H2B^S14P^ peptide was abolished when the cyclin D1 ΔE mutant was introduced (Fig. 2C, D).

We conducted a structural analysis to define the mechanism by which cyclin D1 interacts with H2B. Because the available X-ray and cryo-EM structures of human cyclin D1 lack the E-region we used the AlphaFold generated human cyclin D1 (AF-P25322-F1) structural model [46] with the NMR H2B coordinates PDB ID: 2VQ and deployed the CABS-dock server. Our approach allowed us to independently identify the cyclin D1 binding site of the unstructured tail of histone H2B residues containing S14P. Since the length of the histone tail that interacts with cyclin D1 is unknown, we performed docking using two different length peptides of the H2B unstructured tail, both of which contain S14P. The CABS-dock server placed either of the 2 longer peptides in precisely the same location on the surface of cyclin D1 (Fig. 2E, F). The cyclin D1 binding pocket predicted for H2B is formed by helices α2, α6, and α9, located away from the CDK4/6 interacting site, spanning the entire length of the cyclin D1 molecule (Fig. 2G). The pocket is highly negatively charged, ideal for binding to the positively charged H2B tail. The cyclin D1 residues E74, E76, and E162 are predicted to be likely involved in binding to the K25, R26, and R30 of the H2B C-terminal part of the tail. The cyclin D1 residues E66 and E69 most likely engage K17 and K21, which form part of the center of the H2B tail. The N-terminal portion of the H2B tail forms a hairpin-like structure with Ser14P at the tip of this hairpin. The H2B tail hairpin inserts itself into a cavity formed by helix α9, the E-region, and the following connecting loop (Fig. 2G). In this cavity, H2B residues K8, K9, and K13 most likely engage cyclin D1 residues Q261, Q264, and D282. These interactions place S14P directly in coordinating distance from the E-region glutamates E274 and E278 (Fig. 2H).

### Combinatorial modifications of histones govern cyclin D1 binding

**Fig. 3:**
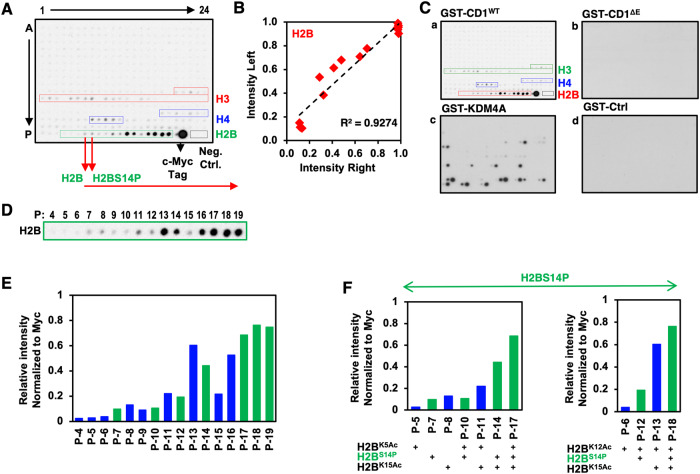
Cyclin D1 association with histones is governed by phosphorylation (H2B^S14^). **A** Representative example of histone array consisting of 384 unique histone modification combinations in duplicate. The array includes up to four different modifications on the same 19mer peptide. For the individual modifications, please see Supplemental Table 3. The Y axis is labeled alphabetically for a given histone in each row, with modifications labeled numerically on the X axis. As each spot is arrayed in duplicate, representative examples of cyclin D1 binding to modified histones as tested by histone-modification arrays and analyzed by active Motif software are shown. **B** Binding is representative of the average positive intensity over the average negative intensity of each modification. The correlation between densitometric analysis of duplicate interactions for each interaction from two separate arrays is shown as intensity left vs. intensity right. The intensity is highly reproducible between duplicate binding assessments (R^2^ = 0.927). (P refers to the designation of the column on the array (H2B), and the number refers to the numerical labeling of the row). **C** Representative examples of histone arrays (384 unique histone modifications on the same 19mer peptide), with binding shown to GST-cyclin D1 (GST-CD1^WT^), GST-CD1^ΔE^, GST-KDM4A or GST-ctrl fusion proteins. **D** Representative and (**E**) quantitated binding, representing average positive intensity over the average negative intensity of each modification for H2B 19mer peptide. **F** Graphical quantification of cyclin D1 binding to post-translationally modified H2B^S14^ 19mer peptide.

To assess the binding of cyclin D1 to post-translationally modified histones in an unbiased manner, the cyclin D1 and KDM4A fusion proteins were assessed for interaction with histone arrays. The arrays consist of 384 unique histone modification combinations in duplicate, including up to four different modifications on the same 19mer peptide (Fig. 3A). As each spot is arrayed in duplicate, binding intensity analysis was conducted for each of the duplicate spots to compare relative binding intensity (left *vs*. right) which demonstrated a highly significant correlation for binding of each duplicate spot (R^2^ = 0.93) as one indicator of binding specificity (the red dotted line in Fig. 3B). The GST-cyclin D1 protein bound modified histones (H2B > H4 > H3), and the GST-cyclin D1ΔE protein showed substantially reduced binding (Fig. 3Ca–c). The pattern of binding by KDM4A resembled previously described preferences (Fig. 3Cd) [47]. To further interrogate the repertoire of post-translational histone modifications that may determine binding to cyclin D1, we quantitated interactions from multiplicate analysis of the 384 unique histone modification arrays on the 19mer peptides (Fig. 3D–I). H2B binding to cyclin D1 was enhanced by the additional modification of H2B^S14^ (Fig. 3D). This enhancement of binding was observed for H2B^K5Ac^, H2B^K12Ac^ and for H2B^K15Ac^ (Fig. 3F). Cyclin D1 recognized and showed enhanced binding for H2B that had been modified by multiple clusters of modifications (H2B^K5Ac^ *vs*. H2B^K5Ac-S14P^, H2B^K12Ac^ *vs*. H2B^K12Ac-S14P^, H2B^K15Ac^ *vs*. H2B^K15Ac-S14P^) (Fig. 3F).

### An intrinsically disordered domain (IDD) in cyclin D1

**Fig. 4:**
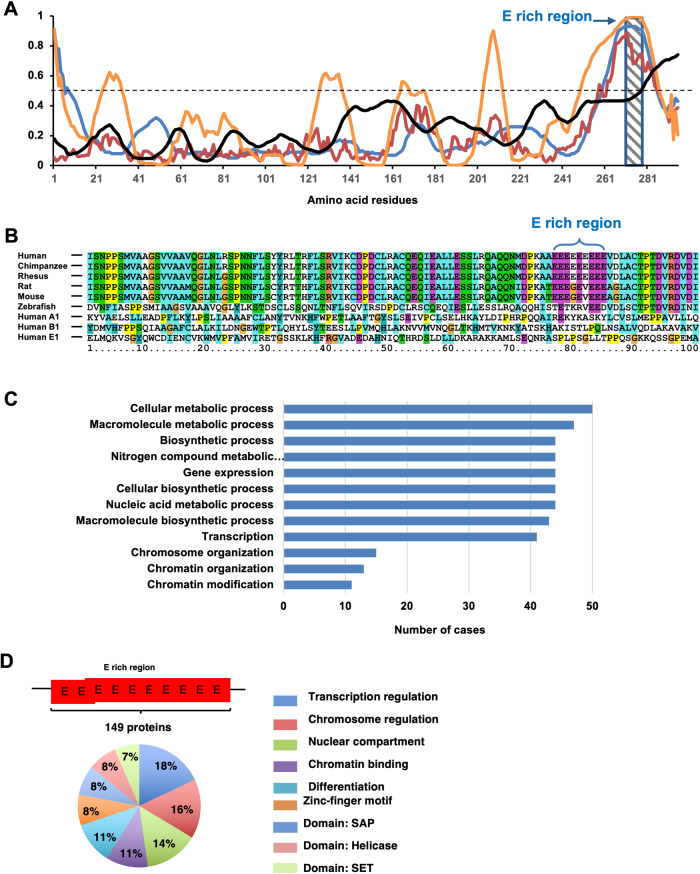
The cyclin D1 carboxyl-terminal domain has intrinsic disorder tendency. **A** Disorder tendency and disordered binding site prediction. PRDos (blue), IUPred long (maroon), PONDR (orange) predictions show that the carboxy-terminal 40 residues (aa 256 - aa295) have a strong tendency to be intrinsically disordered, whereas ANCHOR (black) suggests that last 15 amino acids contain a protein-protein interaction site. **B** Multiple sequence alignment of the last (carboxyl-terminal) 100 amino acids of cyclin D1 from various species and of human cyclin A1, B1 and E1. The E-box motif was found in 148 human proteins by BLASTP, and their enrichment (**C**) and ratio (**D**) in GO Biological Process (BP) categories (“regulation of…”, no. of hits in the given category/148 polyE proteins) is shown.

The strongly acidic nature of the cyclin D1 carboxyl-terminus, suggests this region may be intrinsically disordered. To assess the disorder status of the cyclin D1 carboxyl-terminal region, we applied a range of disorder predictors, including PrDOS, IUPred, and PONDR (Fig. 4A). The prediction results obtained showed a strong correlation with the consensus prediction taken from MobiDB 4.0 (Fig. 4A). A broad range of prediction models derived “Disorder consensus” for the region (Fig. S1A). The predictors rely on different principles, and their consensus is a highly dependent indication of the fundamental structural status of the protein. The predictors agree that most of the cyclin D1 sequence encodes for a structured protein (aa1-aa255, corresponding to a carboxyl-terminal and an amino-terminal domain), whereas the carboxyl-terminal 40 residues (aa256-aa295) have a strong tendency to be intrinsically disordered. Considering the training sets of the predictors relying on examples of IDPs/IDRs, we can be sure that this region does not fluctuate around an equilibrium conformation (that is, it is not “flexible”). Rather it is disordered and can assume a broad ensemble of conformations most appropriate for potentially adapting to diverse binding partners. This is particularly true for the E-rich region. The last carboxyl-terminal 15 residues appear as a down-peak on the disordered pattern (Fig. 4A) with alternating acidic and aliphatic residues, and its behavior of predicted disorder and local hydrophobicity is characteristic of IDP binding motifs. In agreement, this region gives a very strong signal by the ANCHOR predictor (Fig. 4A) developed to recognize specific protein recognition sites embedded in IDPs/IDRs [48].

Within the carboxyl-terminal 100 amino acids, the carboxyl-terminal hydrophobic stretch of the last 15 residues (VDLACTPTDVRDVDI) is the most conserved element in cyclin D1, which, along with the poly E region, is partially present in the remotely related zebrafish cyclin D1 (Fig. 4B). The E-rich region is specific to cyclin D1. It is not found in the closest homologs cyclin D2 and D3 (Fig. S1B). Otherwise, little conservation with distant species is seen in the rest of its C-terminal 100 residues, arguing strongly for the functional importance of the polyE region and the last 15 residues of cyclin D1. Interestingly, the full polyE repeat appears to have arisen in mammals, which fits into the general evolutionary trend that repeat expansion generating homopolymeric amino acid runs is a vehicle of late evolutionary innovation, as their level is much higher in eukaryotes than in prokaryotes and much higher in mammals than in lower vertebrates [49]. The observed conservation pattern of the polyE region in the CTD of cyclin D1 is consistent with this finding. It suggests that the functionality we report is a sign of an evolutionary functionalization event that occurred at the dawn of mammals.

Using the protein BLAST (BLASTP) data mining approach, the cyclin D1 E-rich motif, at least the minimum 9 glutamate residues, can be found in 148 unrelated proteins (Table S1), many of which have chromatin-related functions (Fig. 4C, D). By scrambling the sequences of these hits, we determined the expected distribution of poly E segments in them. The observed 9 residue-long or longer stretches occur highly significantly more frequently (Fig. S2, U-test *p*-value < 1E^-70^) than expected by chance (see *Materials and Methods* for details), which points to the functionality of this motif. In accord, we found that ~50% of these proteins containing E-rich regions participate in chromatin binding, transcriptional regulation, or chromosomal organization. This conclusion is supported by a detailed analysis for GO Biological Process (BP), Molecular Function (MF), and Cellular Component (CC) ontologies, determining enrichments with reference to the whole human genome (Fig. 4C), as shown by hypergeometric test to be highly significant (Fig. S3).

### Cyclin D1 governs Top2A abundance, transcription, and co-activator recruitment to the Top2A promoter

**Fig. 5:**
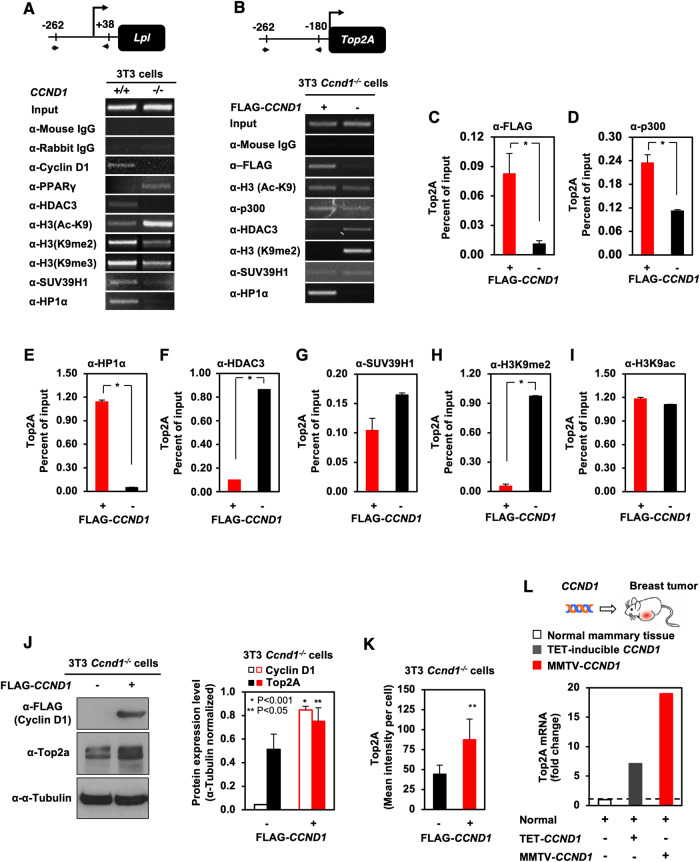
Cyclin D1 recruits distinct chromatin remodeling complexes to repressed (LPL) vs. activated (Top2A) promoters. Endogenous murine LPL ChIP assay (**A**) and Top2A ChIP assays (**B**−**I**) with antibodies directed to target proteins, as indicated in the figure. **C**−**I** Recruitment of the indicated proteins to Top2A promoter was determined by ChIP and real-time quantitative PCR (qPCR). All data are mean ± SEM and represent *n* = 3 experiments. *P*-values were determined by the student’s *t*-test. **J** Western blot for Top2α and β tubulin abundance in cyclin D1^-/-^ 3T3 cells rescued with a cyclin D1 expression vector with quantitation of multiplicate experiments. **K** Top2a intensity assessed by immunofluorescent staining in cyclin D1^-/-^ cyclin D1 rescue 3T3 cells with data shown as mean ± SEM for *N* = 5. **L** Relative Top2A mRNA abundance in the mammary gland of Tet-inducible cyclin D1 mammary epithelial cell targeted transgenic mice (7 days of transgenic induction) or MMTV-cyclin D1 transgenic induced mammary gland tumors.

In order to examine the potential role of the E domain in gene transcription we conducted ChIP analysis to assess recruitment of cyclin D1 to transcriptional targets. The lipoprotein lipase (*LPL*) gene is repressed by cyclin D1, as a component of cyclin D1-mediated restraint of lipogenesis [50], via transcriptional mechanisms associated with the recruitment of cyclin D1 in ChIP assays [29, 30]. As a form of positive control, ChIP assays of the *Lpl* promoter in *cyclin D1*^*+/+*^ 3T3 cells were conducted, showing the previously described presence of cyclin D1 in the context of local chromatin, an increased abundance of HDAC3, reduced H3K9 acetylation, the presence of heterochromatin protein 1 alpha (HP1α) and SUV39H1. Furthermore, associated with histone methylase SUV39H1, we identified increased di- and tri-methylation of H3K9 (Fig. 5A).

Prior genome-wide ChIP seq studies identified regions of genes to which cyclin D1 was significantly recruited into chromatin [8]. Cyclin D1 bound to the *Top2A* gene at −262 to −180, a site distinct from the previously characterized cell-cycle control element (−51 to +90) [51]. The *Top2A* gene is a representative example of the chromosomal instability gene (CIN) signature induced by cyclin D1 [8]. ChIP analysis was conducted to characterize the cyclin D1-mediated chromatin-associated complexes recruited to the endogenous *Top2A* gene regulatory region (Fig. 5B). In *cyclin D1*^*-/-*^ 3T3 cells rescued with cyclin D1^WT^, recruitment of cyclin D1 to the murine *Top2A* promoter was associated with increased HP1α, p300 and with reduced abundance of HDAC3 (Fig. 5C–F). A quantitative analysis of multiple experiments demonstrated that cyclin D1 recruitment to the *Top2A* regulatory region (Fig. 5F, *P* = 0.035) was associated with the co-recruitment of p300 and HP1α (Fig. 5D, E, *P* = 0.011 and *P* = 0.029, respectively), reduced recruitment of HDAC3 (Fig. 5F, *P* = 0.009), correlating with substantially reduced H3^K9me2^ (Fig. 5H, *P* = 0.005) and unaltered H3K9 acetylation (Fig. 5I).

In *cyclin D1*^*+/+*^ (*cyclin D1*^*-/-*cyclin D1^) *vs cyclin D1*^*-/-*^ 3T3 cells, Top2A abundance was increased by Western blot (Fig. 5J, *P* = 0.038) and by IHC (Fig. 5K). To define the impact of cyclin D1 on Top2A abundance in vivo, inducible mammary gland-targeted transgenic mice were deployed (Fig. 5L). Transient (7 day) expression of transgenic cyclin D1 in the mammary epithelium is sufficient for the induction of CIN in vivo [8]. The abundance of *Top2A* was induced 7-fold by cyclin D1 expression in the mammary gland of transgenic mice and *Top2A* expression was induced ~19-fold in MMTV-cyclin D1 tumors (Fig. 5L). The rescue of cyclin D1 into *cyclin D1*^*-/-*^ cells in this system provides cyclin D1 within the physiological range [8], and the tet-inducible cyclin D1 transgenic mice express cyclin D1 at levels observed in mammary tumors [8, 9].

Analysis of *Top2A, Top2B*, and *cyclin D1* expression in 2254 human breast cancer samples [8] showed *cyclin D1*, and *TOP2A* co-expression was associated with ERα^+^ tumors (Fig. S4A, B). Furthermore, scatter plot of cyclin D1 and Top2A abundance by breast cancer subtype depicts the significant correlation between cyclin D1 and Top2A in luminal B (*r* = 0.08. *p* = 0.24) and normal-like (*r* = 0.29, *p* = 1.2e^-05^) breast cancers (Fig. S4D, F).

### A cyclin D1 carboxyl terminal glutamate-rich motif is required for recruitment to the *Top2A* promoter and DNA damage repair signaling, but not to induce cellular polarity and migration

**Fig. 6:**
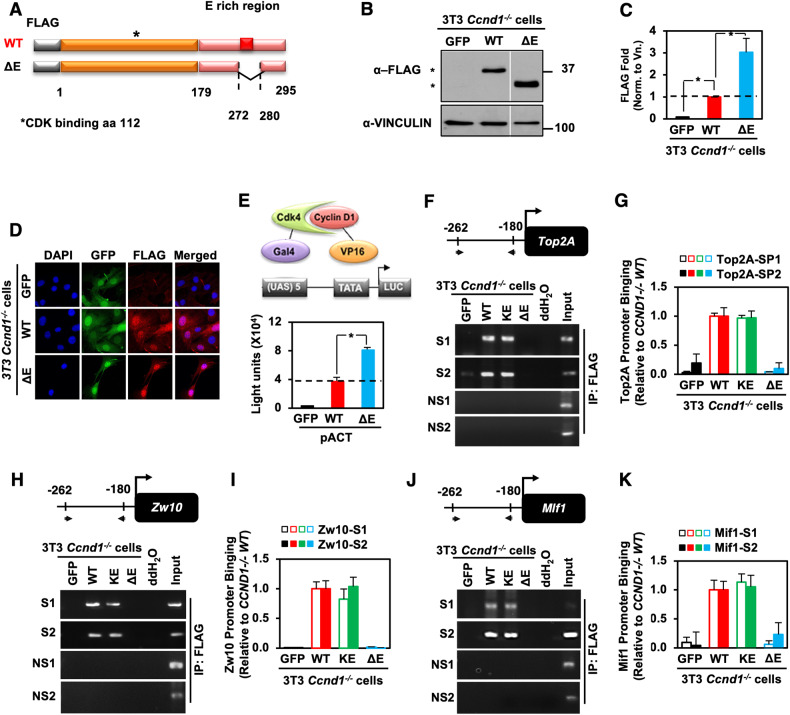
The cyclin D1 Glutamic acid (E)-rich region is required for Top2A promoter occupancy. **A** Schematic representation of cyclin D1^wt^ and cyclin D1^ΔE^ mutant with internal deletion of the E-rich region (red), FLAG tag (gray), C terminal region (pink), and remaining (orange) region. **B** Western blot detection of the FLAG-tagged expression vectors and (**C**) densitometric analysis of cyclin D1 proteins shown as mean ± SEM for *N* = 5 separate experiments (*P* < 0.05). **D** Immunofluorescence staining of the FLAG epitope. GFP (Green) from the vector IRES, DAPI (Blue), and FLAG (Red). **E** Mammalian 2-hybrid interaction of cyclin D1^wt^ and cyclin D1^ΔE^. shown as mean ± SEM for *N* > 5 separate transfections conducted in MCF7 cells. **F** ChIP assays of cyclin D1^-/-^ 3T3 cells rescued with the indicated FLAG-tagged cyclin D1 proteins conducted with the Top2A promoter with oligonucleotide pairs that were either specific (S) to the region identified in ChIP-seq or non-specific (NS) targeted to a region not identified in ChIP-seq as binding to cyclin D1. PCR and (**G**) detected occupancy. Quantitation of PCR products was shown as mean ± SEM for *N* = 3. **H**, **I** Representative ChIP analysis and quantitation of the PCR products derived from cyclin D1 proteins binding Zw10 or (**J**, **K**) Mlf1 regulatory region.

Because cyclin D1 is recruited to the *Top2A* promoter in ChIP-Seq [8] and ChIP, we deployed the *Top2A* promoter to define the domain(s) of cyclin D1 required for recruitment into the context of local chromatin using expression vectors encoding FLAG-tagged cyclin D1^WT^, and cyclin D1 mutants (Fig. 6A). Western blot demonstrated the greater abundance of the cyclin D1^ΔE^ compared with the cyclin D1^WT^ mutant in cultured cells (Fig. 6B, C). The abundance of the cyclin D1 ΔE mutant was 3-fold greater than cyclin D1^WT^ when normalized to vinculin (Fig. 6B, C, *P* < 0.010). Immunofluorescence analysis of the transduced cells demonstrated similar levels of GFP expression from the retrovirus internal ribosome entry site in transfected cells (Fig. 6D). Cyclin D1 induces cellular polarity and cellular migration [23]. Herein, the reintroduction of cyclin D1^WT^ or the cyclin D1^ΔE^ mutant into *cyclin D1*^*-/-*^ 3T3 cells restored cellular polarity (Fig. S5) and transwell migration (Fig. S6), whereas the mutant of cyclin D1 that is defective in CDK4/6 binding due to a point substitution of K114 (cyclin D1^KE^) and a mutant of cyclin D1 encoding amino acids 179−295 *(*cyclin D1^C4^ mutant) were defective (Fig. S5, 6).

Cyclin D1^WT^ bound CDK4 in mammalian two-hybrid assays in MCF7 breast cancer cells (Fig. 6E, *P* < 0.001) and 293 T cells (Fig. S7). The cyclin D1^ΔE^ mutant showed enhanced CDK4 binding in MCF7 (Fig. 6E, *P* < 0.001) and 293 T cells (Fig. S7C). An expression vector encoding three distinct point mutations within the E region (E278A, E280A, E278/280 A) also maintained enhanced binding to CDK4 (Fig. S7C). Mutation of the CDK4/6 binding site of cyclin D1 (cyclin D1^KE^) abolished binding to CDK4 (Fig. S7A–C). Amino-terminal deletion mutants of cyclin D1 that removed the CDK binding site (C1-C5), abolished interaction with CDK4 (Fig. S7C). The reintroduction of cyclin D1 into *cyclin D1*^*-/-*^ 3T3 cells increased the proportion of cells in the S phase and G_2_/M. Induction of G_2_/M was abolished by the cyclin D1^ΔE^ mutant whereas induction of S phase was maintained (Fig. S8). The cyclin D1 C4 mutant was sufficient to induce the G_2_/M phase but did not induce S phase. These findings are consistent with our model in which the CDK binding and thereby S phase function are distinct and uncoupled from the transcriptional activities through the E domain to induce the expression of genes governing chromosomal instability [8] in a CDK-independent manner [9].

Cyclin D1 enhanced doxorubicin-induced phosphorylation of H2A at Ser^139^ and increased comet tail formation, as previously shown [25], with a reduction upon deletion of the E rich region (Fig. S9A, B). Phosphorylated H2AX (γ-H2AX) is a well-accepted marker of DNA double strand breaks [52]. Upon doxorubicin treatment Western blot analysis showed increased γ-H2AX abundance in cyclin D1^WT^ rescued *cyclin D1*^*-/-*^ MEFs at passage 20 (p20) (Fig. S9C) and passage 21 (p21) (Fig S9D) but not in cyclin D1^ΔE^ rescue cells. These findings suggest that the E region is required for the cyclin D1-mediated augmentation of the DNA damage response. To determine the functional significance of cyclin D1 in Top2A activity, we assessed the role of cyclin D1 in regulating sensitivity to the Top2A inhibitor etoposide, which forms a ternary complex with DNA and the topoisomerase II enzyme (which aids in DNA unwinding), preventing re-ligation of the DNA strands, and by doing so causes DNA strands to break. Because Top2A levels vary throughout the cell cycle, increasing after DNA replication [51], *cyclin D1*^*-/-*^ cells rescued with either cyclin D1^WT^ or the cyclin D1^ΔE^ mutant were treated with mimosine (200 μM, 24 h) to arrest the cells prior to DNA replication [53], and Western blot conducted for Top2A (Fig. S10A). Top2A induction by cyclin D1 was reduced by the cyclin D1^ΔE^ mutant (Fig. S10A). Consistent with the increased abundance of Top2A, the cyclin D1 rescued cells were more sensitive to etoposide (Fig. S10B), with a modest but significant reduction in sensitivity upon deletion of the E domain.

**Fig. 7:**
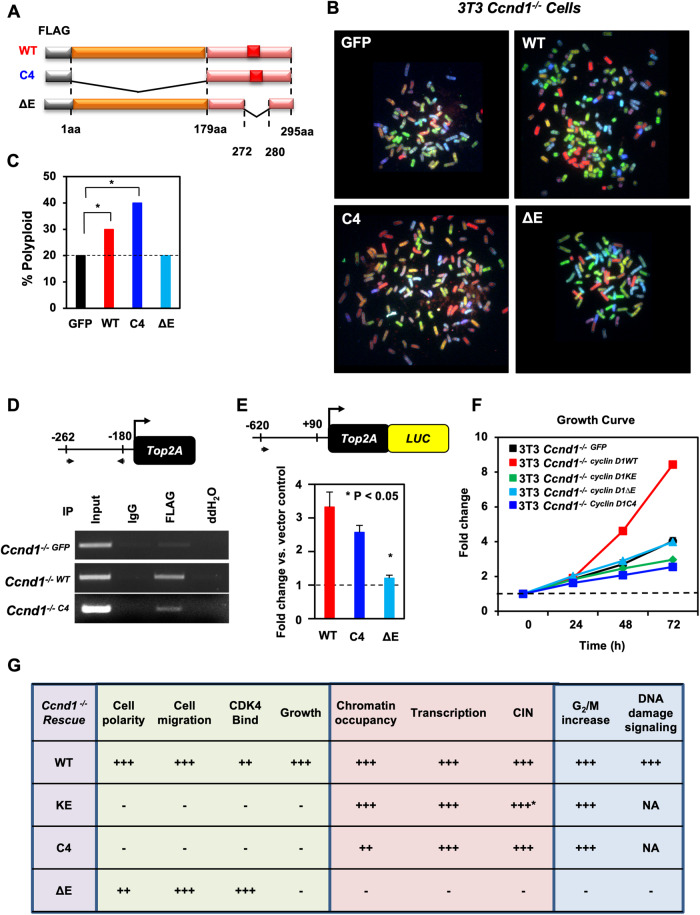
The cyclin D1 carboxyl-terminal domain is necessary and sufficient to induce Top2A promoter activity and polyploidy. **A** Schematic representation of cyclin D1^wt^ and mutants with internal deletion of the E-rich region (red), FLAG tag (gray), C terminal region (pink), and remaining (orange) region. **B** Karyotype determined through SKY analysis for cyclin D1^-/-^ cells rescued with either cyclin D1^wt^ or cyclin D1 mutants with (**C**) mean polyploidy shown. **D** ChIP assays of *cyclin D1*^*-/-*^ 3T3 cells rescued with the indicated FLAG-tagged cyclin D1 proteins were conducted with the Top2A promoter. **E** Top2A promoter luciferase reporter assays are shown as mean ± SEM for *N* > 5 separate transfections. **F** Cellular proliferation assays were conducted in *cyclin D1*^*-/-*^ 3T3 cells, rescued either with cyclin D1^WT^, mutant or control vector. **G** Summary of cyclin D1 mutant properties. The items labeled with * are based on ref. [99].

Analysis was conducted of cyclin D1 binding in chromatin using *cyclin D1*^*-/-*^ 3T3 cells rescued with cyclin D1^WT^ or cyclin D1 mutants. Using two distinct pairs of oligonucleotide probes encompassing the cyclin D1 binding region identified through ChIP-Seq. *Top2A* promoter ChIP analysis demonstrated the recruitment of cyclin D1^WT^ and cyclin D1^KE^ (*P* < 0.010) (Fig. 6F, G). In contrast, the cyclin D1^ΔE^ mutant was not recruited to the *Top2A* promoter (*P* < 0.010) (Fig. 6F, G). Probes directed to regions of the *Top2A* gene, that did not bind cyclin D1 in ChIP-seq did not show binding in ChIP (Fig. 7F, NS). The FLAG antibody that immunoprecipitated cyclin D1 in ChIP from *cyclin D1*^*-/-*Rescue^ cells, did not ChIP the negative control GFP transduced *cyclin D1*^*-/-GFP*^ 3T3 cells (Fig. 6F). Two additional target genes, *Zw10* and *Mlf1*, which like *Top2A* participate in chromosomal stability, and were identified in ChIP-Seq as cyclin D1 target genes [8, 9], bound cyclin D1^WT^ and cyclin D1^KE^ with two distinct sets of oligonucleotide primers but did not bind cyclin D1^ΔE^ in ChIP assays (Fig. 6H–K).

### The C-terminal E domain of cyclin D1 is required to induce chromosomal instability

Previous studies using SKY analysis and gene expression profiling had demonstrated that cyclin D1 re-expression results in CIN, and this action is kinase-independent [8, 9]. To define the domain of cyclin D1 involved in the induction of CIN, we transduced *cyclin D1*^*-/-*^ MEFs with expression vectors encoding cyclin D1^WT^, C4, ΔE, or ctrl and conducted karyotyping (Fig. 7A). SKY was conducted comparing the cyclin D1^WT^, C4 and ΔE mutants *vs*. vector control (GFP) cells. Expression of cyclin D1^WT^, or cyclin D1^C4^ was sufficient for the induction of aneuploidy. Aneuploidy was induced at 72 h by cyclin D1^WT^ (90%, *P* < 0.03) and by cyclin D1^C4^ (100%, *P* < 0.01). In contrast, the cyclin D1^ΔE^ mutant failed to induce aneuploidy compared with control cells (Fig. 7B, C).

We next assessed the transcriptional activation by cyclin D1 using the *Top2A* promoter linked to a luciferase reporter. The site of cyclin D1 binding to *Top2A* in 3T3 cells (−262 to −180), is distinct from the previously characterized cell-cycle control element (−51 to +90) [51]. Cyclin D1^WT^ and cyclin D1^C4^ bound to the *Top2A* promoter in ChIP (Fig. 3D) and induced activity of the *Top2A* promoter 3.5-fold (Fig. 7D). The cyclin D1^ΔE^ mutant that failed to bind the *Top2A* promoter in ChIP assays also failed to induce the *Top2A* promoter luciferase reporter gene (Fig. 7E). Cyclin D1^WT^ increased proliferation 2-fold compared with the empty vector (Fig. 7F). The cyclin D1^KE^, cyclin D1^C4^, and cyclin D1^ΔE^ mutant failed to promote the growth of 3T3 *cyclin D1*^*-/-*^ rescued cells compared to cyclin D1^WT^ (Fig. 7F). Thus, induction of aneuploidy, chromatin recruitment, *Top2A* promoter induction and the induction of cellular proliferation by cyclin D1 requires the poly E region (Fig. 7G). γH2AX is recruited into chromatin in response to signaling stimuli including NMDA [54–57]. Consistent with a model in which cyclin D1 is recruited into chromatin at sites of active DNA damage, ChIP-Seq analysis showed coincident binding for cyclin D1 at ~14% of γH2AX binding sites (Fig. S11A), with several gene targets involved in chromatin structure function and chromosomal instability (Fig. S11B).

These studies suggest a model in which cyclin D1 initiates a DNA damage response, characterized by the induction of γH2AX. Cyclin D1 is known to be recruited to sites of DNA damage with γH2AX [24–26], and such sites co-localize with H2B^Ser14^ [27]. Cyclin D1 associated with high affinity to H2B^S14P^ via the E domain. Collectively these studies identify the E region as an intrinsically disordered domain (IDD) that participates in both the induction of the DNA damage response and transcriptional regulation.

## Discussion

The current studies extend our current understanding of the molecular mechanisms by which cyclin D1 mediates the transcriptional activity. First, these studies identified the minimal E region as required for cyclin D1 binding in chromatin to target genes identified in ChIP-seq (*Top2a, Zw10, Mlf1*). The region of cyclin D1 identified herein is distinct from the region binding directly to TFs, the basal transcription apparatus, or co-activators (AIB1, p300) [32, 58]. Second, we show that the E region is necessary for transcriptional induction by cyclin D1, aneuploidy induction, and the DNA damage response. Deletion of the E region did not affect cyclin D1-mediated induction of polarity, cellular migration, or binding to CDK4 in mammalian two-hybrid analysis in multiple cell types. Third, we show that the carboxyl-terminus is an IDD, particularly at the E region, with features of a protein binding interface seen in other IDD. Fourth, the E region was required for interaction with histone (H2B^S14P^), and in MST, the affinity to H2B^Ser14P^ was similar to that seen with other IDR interactions. Finally, we showed that the E region is present in 148 proteins, many of which are involved in chromosomal function. Cyclin D1 binds in chromatin to induce the transcription of genes governing CIN. As the deletion of the cyclin D1 E domain abolished both the recruitment of cyclin D1 into chromatin and CIN, we propose that the loss of transcriptional function is responsible for the loss of CIN.

Herein the cyclin D1 E region participated in both the DNA damage response and gene transcription, consistent with findings documenting the co-dependence of these processes for particular target genes [54, 56, 59, 60] (reviewed in [61]). Our analysis of cyclin D1 target genes identified in ChIP-Seq [8, 9] showed cyclin D1^WT^ but not cyclin D1^ΔE^ bound in ChIP assays to a variety of target promoters and augmented the DNA damage response. Double-stranded breaks facilitate gene induction mediated by nuclear receptors [60, 62, 63], heat shock, serum stimulation [54], and behavioral responses [55, 57]. TOPIIA (Top2α and Top2β) induce transient double-strand breaks and dissipate torsional stress by relaxing DNA supercoiling [61]. In cyclin D1 ChIP seq the most enriched sites for binding were CTCF sites [8, 9, 32]. CTCF sites are highly enriched for both γH2AX and Top2β within common genomic regions [56]. γH2AX is mainly confined to actively transcribed genes and their downstream regions [57]. Although the precise mechanisms remain to be determined, the induction of gene expression upon DNA damage may involve Top2β-mediated dissolution of CTCF-mediated restraint of enhancer-promoter interactions [56].

Cyclin D1 is recruited to sites of DNA damage [23–25, 64] and previous studies comparing the cyclin D1a and cyclin D1b isoform [25, 65], implicated the unique C-terminal sequence of cyclin D1a in the induction of the DNA damage response. Herein, cyclin D1 was recruited to H2B^S14P^ via the E domain. H2B^S14P^ co-localizes with γH2AX as the primary double-strand break marker in DNA damage foci [27]. Cyclin D1 bound the H2B^S14P^ peptide with significantly higher affinity than H2B^S14^ (Kd = 2.4 μM *vs*. 64 μM) and deletion of the E-rich region (cyclin D1^ΔE^) abrogated binding to either of the peptides. The Kd of cyclin D1 binding to H2B^S14P^ is similar to that of the 14-3-3 protein for H3^S10P^ which ranged from 25-to 92 μM in two different studies [66, 67]. The strength of the cyclin D1/phospho-H2B interaction is comparable to those between other chromatin binding modules and their respective histone modifications, as the binding affinity of methylated H3 tail peptides to chromodomains of HP1 [68] and chromomethylase [69], ranges from 10 to 100 μM. In previous studies of IDD and fuzzy interaction motifs, multiple low-affinity interactions in the pM to μM range [70, 71], are necessary and contribute to the binding specificity of these motifs [71–73]. In our MST experimental protocol, the protein is labeled, therefore the mobility changes, induced by the binding of a small peptide assessed by amplitude of the changes in F_norm,_ are small [74, 75]. The altered directionality of the F_norm_ changes with H2B^S14P^ (Fig. 6G) likely reflects a different mode of binding, as previously described [76]. The peptide concentrations used in MST with cyclin D1^ΔE^ were considerably higher than with cyclin D1^WT^, resulting in slightly higher instability of the MST signal and higher scattering.

The C-terminal region was predicted herein to be intrinsically disordered, with predicted disorder scores dropping toward ordered values in the last 10−15 residues (VDLACTPTDVRDVDI), and strong interaction potential was predicted by ANCHOR [48]. Such patterns are often seen at short (linear) motifs that mediate protein-protein interactions in IDPs/IDRs [77]. It is highly plausible that these prediction tools capture the recently identified degron motif [78]. IDRs are often involved in molecular recognition and protein-protein interaction events due to their large accessible surface area and inherent flexibility [39]. IDPs/IDRs abound in homopolymeric runs of amino acids, and amino acid repeats often mediate their functional interactions [79]. The absolute sequence conservation of the cyclin D1 carboxyl-terminal hydrophobic region among different species underscores the functional importance of this region. Such short motifs of IDPs/IDRs often undergo induced folding (disorder-to-order transition) upon partner binding [41], which might be the case with the binding motif of cyclin D1. The disorder pattern, its hydrophobic character and evolutionary conservation corroborate the experimental observations that the carboxyl-terminal acidic region is involved in mediating the interaction functions of cyclin D1.

IDD interactions can be mediated by post-translational modifications including phosphorylation and acetylation [80]. For example, the IDD identified within CBP and p53 involve transient post-translational modifications of their substrates [81] and the Zinc finger protein 106, upon acetylation by CBP, interacts with an IDD within CBP [82]. Based on the precedent of other IDD behaviors, the impact of histone phosphorylation is predicted to govern folding to a structured state promoting protein-protein interactions [83–85], rather than charge change, which is a relatively trivial difference (pI Cyclin D1: 4.97, pI Cyclin D1 CTD: 3.79, *vs*. pI H3.1: 11.13), as a single phosphorylation event adds 1.5 negative charges to H3.1 (pI H3.1-phospho: 10.87). The Kd values for the association of multiply acetylated H4 tails and hTAFII250 double bromodomain are between 1 and 50 μM [86]. For example, the binding of folded domains, such as SH2 domains, to phosphorylated residues (pTyr), the added negative charge(s) of the phosphate group, results in binding in a dedicated groove, as opposed to the rather unspecific interaction of the unmodified peptide.

The emerging view is that nucleosomes can cluster into a dynamic, fuzzy phase [87] mediated by promiscuous, dynamic interactions of the disordered appendages of core and linker histones and other proteins. Some IDPs can exhibit relatively compact structures such as polyglutamines [88]. These interactions are referred to as “fuzzy” as the weak and transient complexes confer specificity without a structured state [43, 89]. Such an organization has also been observed in the heterochromatin, mediated by the interaction of methylated H3K9 and Heterochromatin protein 1 (HP1) [90]. Our results on the specific and modification-dependent interaction of the disordered region of cyclin D1 and histone tails is also of relevance in light of recent observations that interactions mediated by IDPs/IDRs in chromatin underlie the formation of a liquid-like state, in which nucleosomes are organized in a dynamic, condensed fashion [91]. Such condensates, in general, can form by liquid-liquid phase separation mediated by multivalent interactions of disordered proteins [44]. The E-rich motif is conserved with 148 proteins, many of which are involved in chromatin reorganization. Future studies will be directed to the role of these IDD-containing proteins in chromatin reorganization.

## Materials and methods

### Cell Culture, plasmids, chemicals, and luciferase reporter assays

The HEK293T cell line was obtained from ATCC (Manassas, VA) in the early 2000s. *Ccnd1*^*+/+*^*, Ccnd1*^*-/-*^ *or Ccnd1*^*-/-*^ *cyclin D1* rescued mouse 3T3 fibroblasts were generated from the mouse in this lab [8]. The cells were maintained in Dulbecco’s modified Eagle’s media (DMEM) supplemented with 10% FBS, 2 mmol/L glutamine, 100 units/mL penicillin, and 100 μg/mL streptomycin in a humidified atmosphere containing 5% CO_2_ at 37 °C. The early passages of the cells were stored. Mycoplasma contamination was tested using the Universal Mycoplasma Detection Kit from ATCC to ensure the absence of mycoplasma contamination. The cells thawed from low passage stocks were used within one month of the initial thaw. During the experiments, the morphology of all cell lines was routinely checked under a phase contrast microscope. All of the newly revived cells were treated with Plasmocin (InvivoGen), and any mycoplasma contamination was determined with Hoechst 33258 staining under a high magnification fluorescent microscope routinely.

The mouse stem cell virus-internal ribosome entry site-green fluorescent protein retrovirus vectors, pMSCV-IRES-GFP, and the ecotropic, packaging vector, pSV-ψ-E-MLV and infection methods were as described previously [50]. The cDNA encoding cyclin D1 wt, N1 (aa1-aa287), C4 (aa179-aa295), and deletion of the E-rich motif (ΔE) (aa272-aa280) were generated by PCR. EcoR1 and *Bam HI* restriction sites were introduced via PCR primers. The PCR products were cloned into pMSCV-IRES-GFP vector. The GST expression plasmids were generated using either full length cyclin D1 cDNA, or cyclin D1 ΔE mutant. These fragments were subcloned into the *BamHI* and *EcoR1* sites of pGEX-4T-3 vector (Amersham). For the cloning of the VP16-cyclin D1 constructs the cDNA encoding D1 wt, N1 (aa1-aa287), C4 (aa179-aa295) and deletion of E-rich motif (aa272-aa280) were generated by PCR. *BamHI* and *XbaI* restriction sites were introduced via the primers. The PCR products were cloned into pACT vector (Promega). The human *TOP2A* promoter linked to a luciferase reporter gene was previously described [92] and the data were normalized through co-transfection of a renilla luciferase reporter plasmid (1 ng). Mimosine was from Santa Cruz Biotechnology, and doxorubicin was from Sigma.

MSCV retroviruses were prepared by transient co-transfection with helper virus into 293 T cells, using calcium phosphate precipitation. The retroviral supernatants were harvested 48 h after transfection and filtered through a 0.45-μm filter. *Ccnd1*^-/-^ 3T3 cells were incubated with fresh retroviral supernatants in the presence of 8 μg/ml Polybrene for 24 h, cultured for an additional 4 days, and then subjected to fluorescence-activated cell sorting (FACS) (FACStar Plus; BD Biosciences, San Jose, CA) to select for cells expressing GFP. GFP^+^ cells were subsequently analyzed. *Ccnd1*^*-/-*^ 3T3 cells were rescued with cyclin D1 protein expression vectors with FLAG-tagged cyclin D1 wild type (WT) or the mutants; amino- and carboxyl-terminal deletions (C4 and N1, respectively) and E-rich motif internal deletion (ΔE) and compared with cells transduced with an empty vector expressing IRES-GFP as a control. Wortmannin was used at a final concentration of 200 nM for 6 h.

### Animal model

Animal experiments were approved by Thomas Jefferson University’s Institutional Animal Care and Use Committee (IACUC). Eight to ten weeks-old female mice were used for in vivo experiments. All mouse experiments using the MMTV-cyclin D1 transgenic mice and the tetracycline-inducible mammary epithelial cell targeted cyclin D1a transgenic mice (MMTV-rtTA-cyclin D1) in the FVB strain background were conducted following the guidelines for the care and use of laboratory animals at Thomas Jefferson University.

### Spectral karyotyping analysis

For spectral karyotyping (SKY) analysis, fluorescence color images of chromosomes stained by Rhodamine, Texas Red, Cy5, FITC, and Cy5.5 were captured under a Nikon microscope equipped with a spectral cube and Interferometer module. SKY View software (version 1.62), was used to analyze the chromosomal number and structural alterations of chromosomes, including simple balanced translocations, unbalanced (or nonreciprocal) translocations, deletions, and duplications. At least 10 metaphases were analyzed per sample. Statistical significance was calculated using the chi-square test of association (Pearson).

### Western blotting and Immunofluorescence

Whole-cell lysates (50 μg) were separated by 10% SDS-PAGE gel, and the proteins were transferred to nitrocellulose membrane for Western blotting using the following antibodies: anti-FLAG antibody (M2 Aldrich, #F1804, St. Louis, MO), anti-GST (Cell Signaling, #2624, Danvers, MA), anti-β-tubulin (Santa Cruz Biotechnology, #sc-55529, Santa Cruz, CA), anti-Vinculin (Sigma-Aldrich, #V9131 St. Louis, MO), Topoisomerase 2 A (Santa Cruz Biotechnology, #sc-365916, Santa Cruz, CA), Histone 2B Serine 14 P (Santa Cruz Biotechnology, #sc-31671, Santa Cruz, CA), Histone 2B (Santa Cruz Biotechnology, #sc-10808, Santa Cruz, CA), γH2A.X (Cell Signaling, #9718, Danvers, MA) and H2A.X (Cell Signaling, #2595, Danvers, MA).

Immunofluorescence was performed on *Ccnd1*^-/-^ cells transduced with retrovirus expressing cyclin D1 WT, C4, N1, ΔE, or vector control as previously described [8]. The subcellular localization was determined using the anti-FLAG M2-antibody.

### Chromatin immunoprecipitation assay

Chromatin immunoprecipitation (ChIP) analysis was conducted as described [8] following the protocol provided by Upstate Biotechnology (Charlottesville, VA). Chromatin solutions were precipitated overnight at 4 °C with rotation, using 8 μg of anti-FLAG, 4 μg of anti-Cyclin D1 (HD-11, Santa Cruz Biotechnology, Santa Cruz, CA), H3(Ac-K9) (Millipore, #07-352, Billerica, MA), p300, (Santa Cruz Biotechnology, #sc-585X, Santa Cruz, CA), HDAC3 (Abcam, #ab7030, Cambridge, UK), H3(Dimethyl-K9) (Millipore, #1220, Billerica, MA), SUV39H1 (Millipore, #05-615, Billerica, MA) and HP1α (Millipore, #05-685, Billerica, MA). For negative control, rabbit IgG and mouse IgG were immunoprecipitated. ChIP analysis for each immunoprecipitated protein was conducted on the endogenous murine *Top2A* promoter (35 cycles of PCR). ChIP-DNA quantitation was conducted in an Agilent 2100 bioanalyzer (Agilent Technologies, Palo Alto, CA), using Power SYBR Green (AB Biosciences) according to the manufacturer’s guidelines. Equal quantities of ChIP-DNA were used for the real-time PCR quantitation. Ct values were used to calculate the relative fold enrichment (2^-ΔCt^, ΔCt = Ct^input^ - Ct^IgG^). A one-way ANOVA followed by Student’s *t*-test comparison was performed to compare the relative fold enrichment (*n* = 3).

### Comet assays

Neutral pH comet assays were conducted using the Comet- Assay Kit (Trevigen) as previously described [25]. After treatment with doxorubicin or control, cells were harvested and mixed with low-melting temperature agarose. After lysis, electrophoresis was conducted at 1 V/cm for 20 min. Slides were stained with SYBG green dye for 10 min and visualized on a Zeiss LSM 510 META confocal microscope with a Å~20 objective. The relative length and intensity of SYBG green–stained DNA tails to heads was proportional to the amount of DNA damage present in the individual nuclei and was measured by Olive tail moment using OpenComet software **(**http://www.cometbio.org/index.html**)** [93].

### Protein-protein interaction by mammalian two-hybrid reporter assay

Mammalian two-hybrid was conducted following the manufacturer’s instruction (Promega, Madison, WI). The human CDK4 cDNA was cloned into pBIND vector, and the human cyclin D1 cDNA (wild type or mutants) was cloned into pACT vector to generate fusion proteins with the DNA-binding domain of Gal4 (Gal4-CDK4), and the activation domain of VP16 (VP16-cyclin D1), respectively. Gal4-CDK4, VP16-cyclin D1 and pG5-Luc reporter containing five copies of Gal4 binding sites upstream of a minimal TATA box and the firefly luciferase gene (Promega) were co-transfected into MCF-7 or HEK-293T cells with Superfect reagent 48 h after transfection, the cells were lysed, and the amount of luciferase activity was quantitated. Transfection efficiency was normalized using renilla luciferase.

### Peptide and GST pull-down assays

The Peptide pull-down assays were performed following established protocols. Biotin peptides Histone H2B and Histone H2B^S14P^ were customized and purchased from Anaspec. Pull-down assays were performed using bacterial-purified GST-Cyclin D1 WT, Cyclin D1ΔE deletion, GST-KMD4A, and GST control. Equal molarity of GST-tagged proteins was used. 25 μg of the histone peptides were bound to Streptavidin agarose beads in PBS for 30 min at room temperature, and then the beads were washed 3 times with PBS. Cyclin D1^WT^, Cyclin D1^ΔE^, and GST were incubated with the beads for 1 h at room temperature, with constant mixing, followed by 3 washing steps with PBS (https://www.sigmaaldrich.com/US/en/technical-documents/protocol/protein-biology/enzyme-activity-assays/pull-down-assays).

### Surface plasmon resonance

To analyze further the specific interactions between cyclin D1 and histone modifications, we deployed Surface Plasmon Resonance (SPR). SPR between the proteins was measured using surface plasmon resonance measurements on a Bio-Rad ProteOn^TM^ XPR 36 Protein Interaction Array System. Biotinylated histone peptides were immobilized on the surface of a ProteOn™ NLC Sensor Chip (# 1765021). The chip was initialized and equilibrated as suggested by the manufacturer. The buffer used for ligand capture and partner binding was: 20 mM TRIS, 150 mM NaCl, 2 mM EDTA, 0.5% Tween-20, and pH8.0. The peptides were loaded on the chip surface at a concentration of 5 μg/ml with a flow rate of 30 μl/min to a level of 300 RU (response units). Ligand capture and partner binding experiments were carried out at 25 ^o^C. Binding of different proteins was detected using a concentration range between 0.1 and 2 μM (cyclin D1^ΔE^, GST, and Tudor domain) or 1 and 10 μM (cyclin D1^wt^). Flowrate used for binding was 40 μl/min, the association time was 240 s and the dissociation time varied between 560 and 960 s. A lane without ligand added was used as negative control and the resulting baseline curves were subtracted before binding analysis.

### Microscale Thermophoresis (MST) analysis

Binding assays were carried out on a Microscale Thermophoresis (MST) system (Monolith NT. 115 from NanoTemper Technologies, München, Germany) in reaction mixtures containing fluorescently labeled cyclin D1 variants and unlabeled H2B peptides in PBS. Standard-treated capillaries (Cat. No.: MO-K002) were used for measurements. Instrument settings were as follows: First round: LED Power: 10%, MST Power 20%, Before MST: 5 s, MST on: 30 s, After MST: 5 s, Delay: 25 s. The second round had the same settings, but the MST Power was 40%. For the measurements, GST-tagged Cyclin D1^WT^ and GST-tagged Cyclin D1^ΔE^ were fluorescently labeled with an amine-reactive fluorescent dye (Monolith Protein Labeling Kit RED-NHS 2nd Generation, Cat. No.: MO-L011) according to the protocol provided by the manufacturer. Labeled protein concentrations were set to give an initial raw fluorescence between 800 and 1000 counts and were varied between 3.5 μM and 12.5 μM. All experiments were conducted at room temperature. Labeling and MST measurements were carried out in phosphate-buffered saline (PBS, Cat. No.: 524650, Merck KGaA, Darmstadt, Germany). The exact composition was: 140 mM NaCl, 10 mM phosphate buffer, and 3 mM KCl, pH 7.4. Anaspec (Fremont CA, USA) synthesized histone peptides with C-terminal biotin labels. The sequences of the peptides were: Unmodified H2B: PEPAKSAPAPKKGSKKAVTKAQ, Phosphorylated H2B: PEPAKSAPAPKKGpSKKAVTKAQ. Starting peptide concentrations were 15 times higher than the Cyclin D1 concentration in the reaction mixture. Curve fitting and Kd calculations were carried out with the software provided with the instrument (MO Affinity Analysis, Version 2.3). A detailed description of the MST method and the interpretation of the results can be found in Jerabek-Willemsen et al. [76, 94].

### Histone peptide array

The modified histone peptide arrays were purchased from Active Motif (Active Motif, # 13001, Carlsbad, CA) and analyzed as described by the manufacturer’s protocol using the buffer (100 mM KCl, 20 mM HEPES pH 7.5, 1 mM EDTA, 0.1 mM DTT and 10% glycerol). The membranes were incubated with purified protein either from full-length or mutant-expressed GST-Cyclin D1^WT^ or GST-Cyclin D1^ΔE^.

### Protein data mining analysis and prediction of disorder tendency and multiple sequence alignment

BLASTP (National Library of Medicine) was used to retrieve all proteins that contain a minimum of 9 tandem glutamine repeats. The search retrieved 149 hits, including cyclin D1, of which there were 148 unique proteins (and one pseudogene). The 148 unique proteins were inputted into the DAVID functional annotation clustering algorithm to display and cluster similar annotation terms with an enrichment score of >1.5.

Four different disorder prediction algorithms assessed structural disorder: PrDOS [95], IUPred [96], RONN [97] and DISOPRED2 [98]. The results obtained showed a high correlation with the consensus prediction from MobiDB 4.0 (https://mobidb.bio.unipd.it). These predictors rely on different principles and represent different stringencies. Thus their consensus offers exquisite reliability in the disorder status of a (segment) of a polypeptide chain. Briefly, PrDOS is composed of two predictors, one based on local amino acid sequence and another based on template proteins, and is most appropriate for predicting short, disordered segments. IUPred relies on assessing the total pairwise interaction energy encoded in a polypeptide chain and has options for predicting long and short disorder. The “long” option can detect context-independent global disorder of a protein that encompasses at least 30 consecutive residues of predicted disorder. The “short” option is suitable for predicting short, probably context-dependent disordered regions. RONN (Regional Order Neural Network) is a bio-basis function neural network trained on disordered proteins that predicts regions that lack a well-defined 3D structure under native conditions. DISOPRED2 is based on cascaded support vector machine classifiers trained on PSI-BLAST profiles.

Multiple sequence alignments were conducted with the ClustalW server at the EMBL-EBI website (http://www.ebi.ac.uk/Tools/msa/clustalw2/) on sequences downloaded from the UniProt site (http://www.uniprot.org/). A graphic representation of the data was prepared with the ClustalX software.

To evaluate the significance of the enrichment of E-rich region sequences, the length distribution of poly E segments was extracted and compared to random sequences. The 148 protein hits (Table S2) were scrambled 100 times, and the average length distribution of poly E segments was recorded. Mann-Whitney’s U-test indicated that the E-rich region sequences and their scrambled variants have extremely significantly different poly E length distributions, with a *p*-value < 10^−70^, suggesting that the observed distribution (and the presence of a poly E region of 9 consecutive Glu-structure) could not have occurred by chance.

### Cyclin D1-H2B model generation

The human cyclin D1-H2B model was generated using the CABS-dock server (http://biocomp.chem.uw.edu.pl/CABSdock/) [99, 100] without defining a specific H2B binding site of cyclin D1 thus allowing for the software to identify the best fit independently. H2B docking into cyclin D1 structure was carried out in three steps [99, 100]. Briefly, the server first converts the cyclin D1 into a coarse‐grained representation thus simulating the behavior of complex systems. The CABS-dock server uses a Replica Exchange Monte Carlo pseudo‐dynamics with annealing for protein dynamic simulation. The next step involves the placement of H2B in various possible protein binding pockets approximately 20 Å from the cyclin D1 surface. H2B peptides used in docking were randomly selected from a library of 10 generic H2B starting conformations. We selected the default CABS-dock mode, which uses 10 replicas and 20 annealing cycles. The available x-ray or cryo-EM structures of human cyclin D1 lack the E-region so we used the AlphaFold generated CD1 (AF-P25322-F1) and the NMR H2B coordinates PDB ID: 2VQ in our modeling. The docking was carried out with two different length peptides of the H2B tail: PEPAKSAPAPKKGSKKAVTKAQKKDGKKRKRSR, and PEPAKSAPAPKKGSKKAVTKAQKKDGKKRKR, as it is not clear what is the precise length of the H2B tail that binds cyclin D1. Both peptides contain the residue S14 but either lack or include residues C- and N-terminal to it. The CABS-dock server placed both longer peptides in precisely the same location on the surface of cyclin D1. In this study, we selected the best binding mode of H2B from the 10-top scores. The figures were made in PYMOL (The PyMOL Molecular Graphics System, Version 2.0 Schrödinger, LLC) and the electrostatic surface charge generated with APBS [101].

### ChIP-Seq analysis

Cyclin D1 CHIP-Seq data in mouse embryonic fibroblasts is from our previous work (GSE207361) [8]. γH2A.X Chip-Seq data is from Dr. Madabhushi’s published work (GSE61887) [56]. The track of cyclin D1 and γH2A.X in the chip-seq were viewed and captured with Integrated Genome Browser.

### Statistical analysis

The statistical analysis was performed using Prism 4 software (GraphPad Software, San Diego, CA). Differences were considered statistically significant with *P*-values < 0.05.

### Supplementary information

Supplemental figures and tables

## Data Availability

All data generated or analyzed during this study are included in this published article and its supplementary information files.
