# Supplementary material for: A cyclin D1 intrinsically disordered domain accesses modified histone motifs to govern gene transcription
Source: Oncogenesis. 2024 Jan 8;13(1):4. doi: 10.1038/s41389-023-00502-1 (PMC10774418; doi:10.1038/s41389-023-00502-1)
Supplement: Supplementary file 1 — Supplemental figures and tables [file 41389_2023_502_MOESM1_ESM.pdf]

SUPPLEMENTAL MATERIALS

A CYCLIN D1 INTRINSICALLY DISORDED DOMAIN ACCESSES MODIFIED HISTONE MOTIFS TO GOVERN GENE TRANSCRIPTION

Xuanmao Jiao[1,2*], Gabriele Di Sante[1*], Mathew C. Casimiro[1,3], Agnes Tantos[4], Anthony W. Ashton[1,2,5], Zhiping Li[1,2], Yen Quach[2], Dharmendra Bhargava[1], Agnese Di Rocco[1], Claudia Pupo[6], Marco Crosariol[6], Tamas Lazar[7], Peter Tompa[4,7], Chenguang Wang[6], Zuoren Yu[8], Zhao Zhang[1], Kawthar Aldaaysi[2], Ratna Vadlamudi[9], Monica Mann[9], Emmanuel Skordalakes[10], Andrew Kossenkov[10], Yanming Du[1] and Richard G. Pestell[1,2,10**]

SUPPLEMENTAL FIGURES

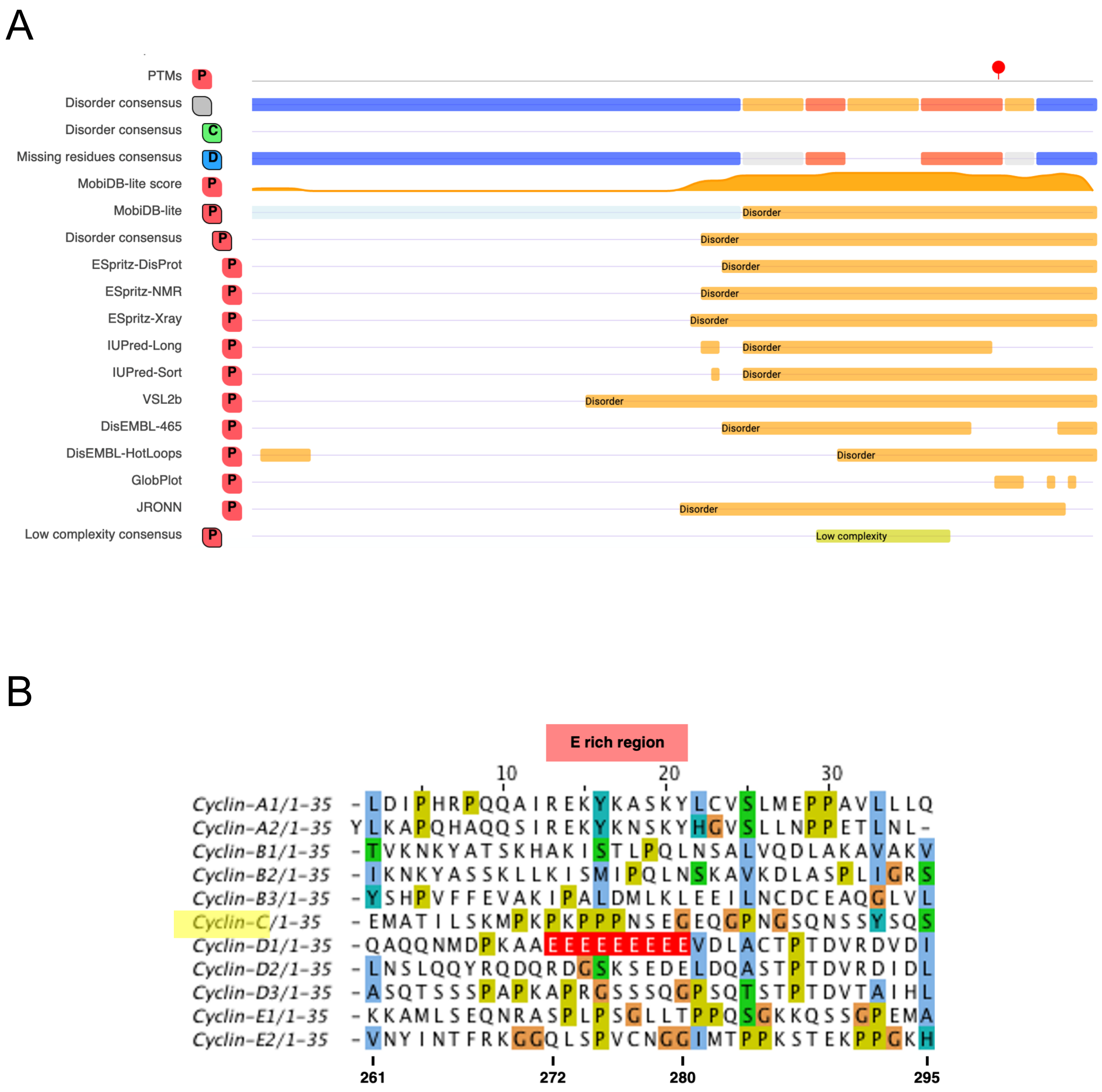

Figure S1. (A). MobiDB consensus prediction of protein disorder. For the consensus prediction of structural disorder in the C-terminal domain, the MobiDB consensus predictor was used for the last 81 residues (aa.215-295) of human cyclin D1. Consensus predicted disorder derived from a broad range of predictions is seen as "Disorder consensus". For the interactive view of the full-length prediction results visit the MobiDb 4.0 website: https://mobidb.bio.unipd.it/P24385. **(B). The cyclin D1 carboxyl-terminal domain has an intrinsically disordered tendency.** Multiple sequence alignment of the last 40 carboxyl-terminal amino acids of cyclin D1 with human cyclin A1, B1 and E1 and D type cyclins. The E box is unique to cyclin D1.

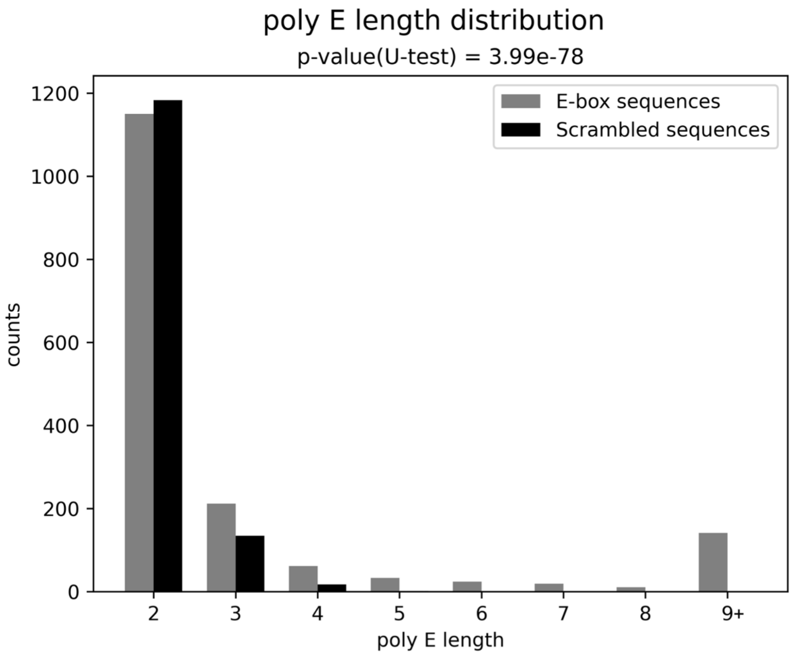

Figure S2. The length distribution of poly E segments of E-box proteins compared to those of randomized sequences. The Mann-Whitney's U-test indicates that the E-box sequences and their scrambled variants have significantly different poly E length distributions with p-value $< 1E^{-70}$. Although length distributions were calculated for poly E lengths between $E^{(2)}$-$E^{(32)}$ consecutive E letters, bins $E^{(9)}$-$E^{(32)}$ were aggregated in bin '9+'.

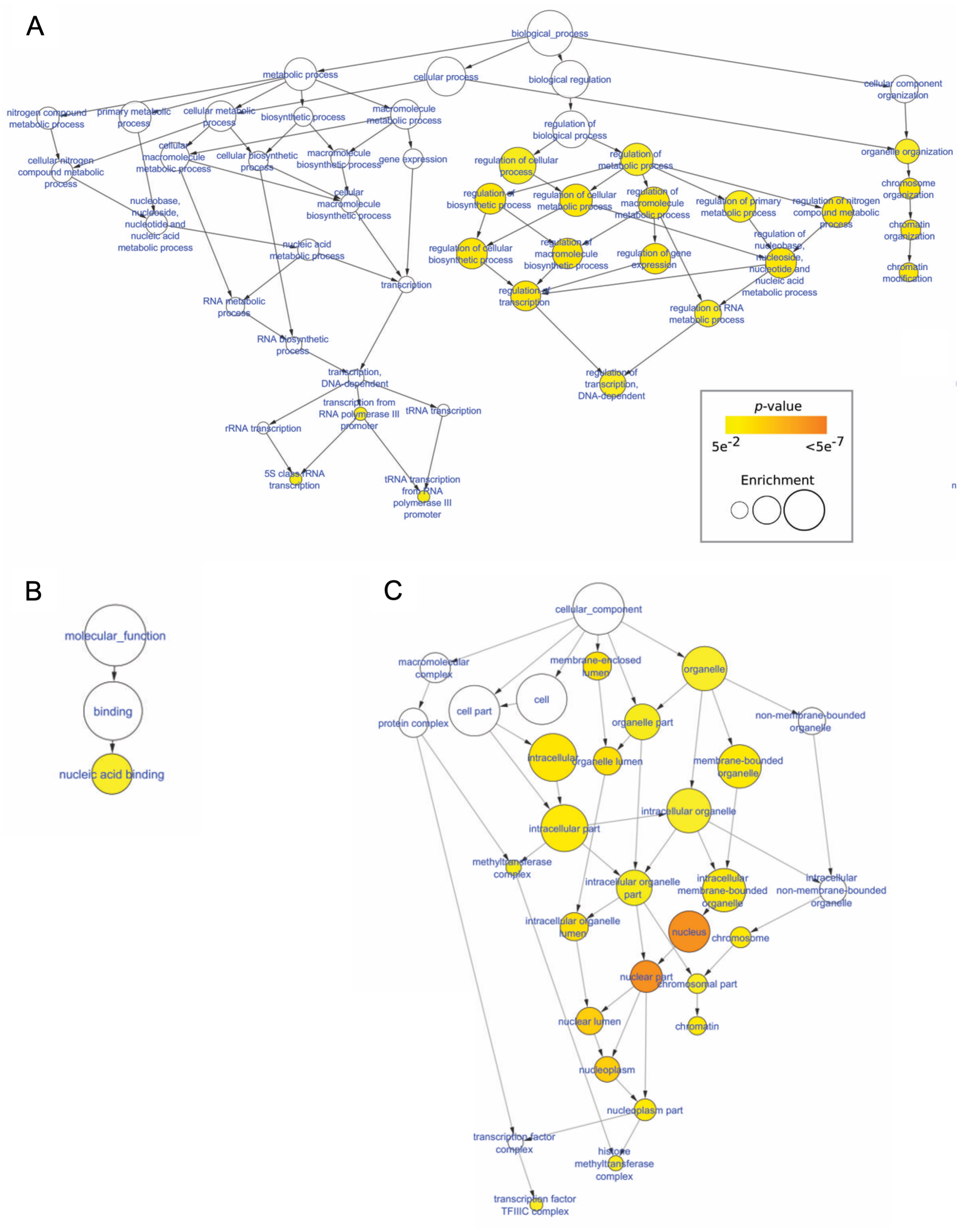
A
biological_process
metabolic process
cellular process
biological regulation
cellular component organization
nitrogen compound metabolic process
primary metabolic process
cellular metabolic process
biosynthetic process
macromolecule metabolic process
regulation of biological process
organelle organization
cellular nitrogen compound metabolic process
cellular macromolecule metabolic process
cellular biosynthetic process
macromolecule biosynthetic process
gene expression
regulation of cellular process
regulation of metabolic process
chromosome organization
nucleobase, nucleoside, nucleotide and nucleic acid metabolic process
cellular macromolecule biosynthetic process
regulation of biosynthetic process
regulation of cellular metabolic process
regulation of macromolecule metabolic process
regulation of primary metabolic process
regulation of nitrogen compound metabolic process
chromatin organization
nucleic acid metabolic process
regulation of cellular biosynthetic process
regulation of macromolecule biosynthetic process
regulation of gene expression
regulation of nucleobase, nucleoside, nucleotide and nucleic acid metabolic process
chromatin modification
transcription
RNA metabolic process
regulation of transcription
regulation of RNA metabolic process
RNA biosynthetic process
transcription, DNA-dependent
regulation of transcription, DNA-dependent
transcription from RNA polymerase III promoter
rRNA transcription
tRNA transcription
5S class rRNA transcription
tRNA transcription from RNA polymerase III promoter
p-value
5e-2
<5e-7
Enrichment
B
molecular_function
binding
nucleic acid binding
C
cellular_component
macromolecular complex
membrane-enclosed lumen
organelle
cell
cell part
protein complex
organelle part
non-membrane-bounded organelle
intracellular
organelle lumen
membrane-bounded organelle
intracellular organelle
intracellular part
methyltransferase complex
intracellular organelle part
intracellular membrane-bounded organelle
intracellular non-membrane-bounded organelle
intracellular organelle lumen
nucleus
chromosome
nuclear part
chromosomal part
nuclear lumen
chromatin
nucleoplasm
nucleoplasm part
transcription factor complex
histone methyltransferase complex
transcription factor TFIIIC complex

Figure S3. Enrichment in GO categories of poly E-containing human proteins. Enrichment analysis of the 148 polyE-containing human proteins (Table S2) was carried out with the BINGO application for Cytoscape with all possible evidence codes, for the Biological Process (BP, panel A), Molecular Function (MF, panel B) and Cellular Component (CC, panel C) GO ontologies. Enrichments were computed using as reference to the whole *Homo sapiens* genome. The statistical significance of the computed enrichment was evaluated using the hypergeometric test with Benjamini and Hochberg false discovery rate (FDR) multiple testing correction. The size of the circles in the graph indicates the enrichment in a particular GO term, and its color the corrected p-value (see inset).

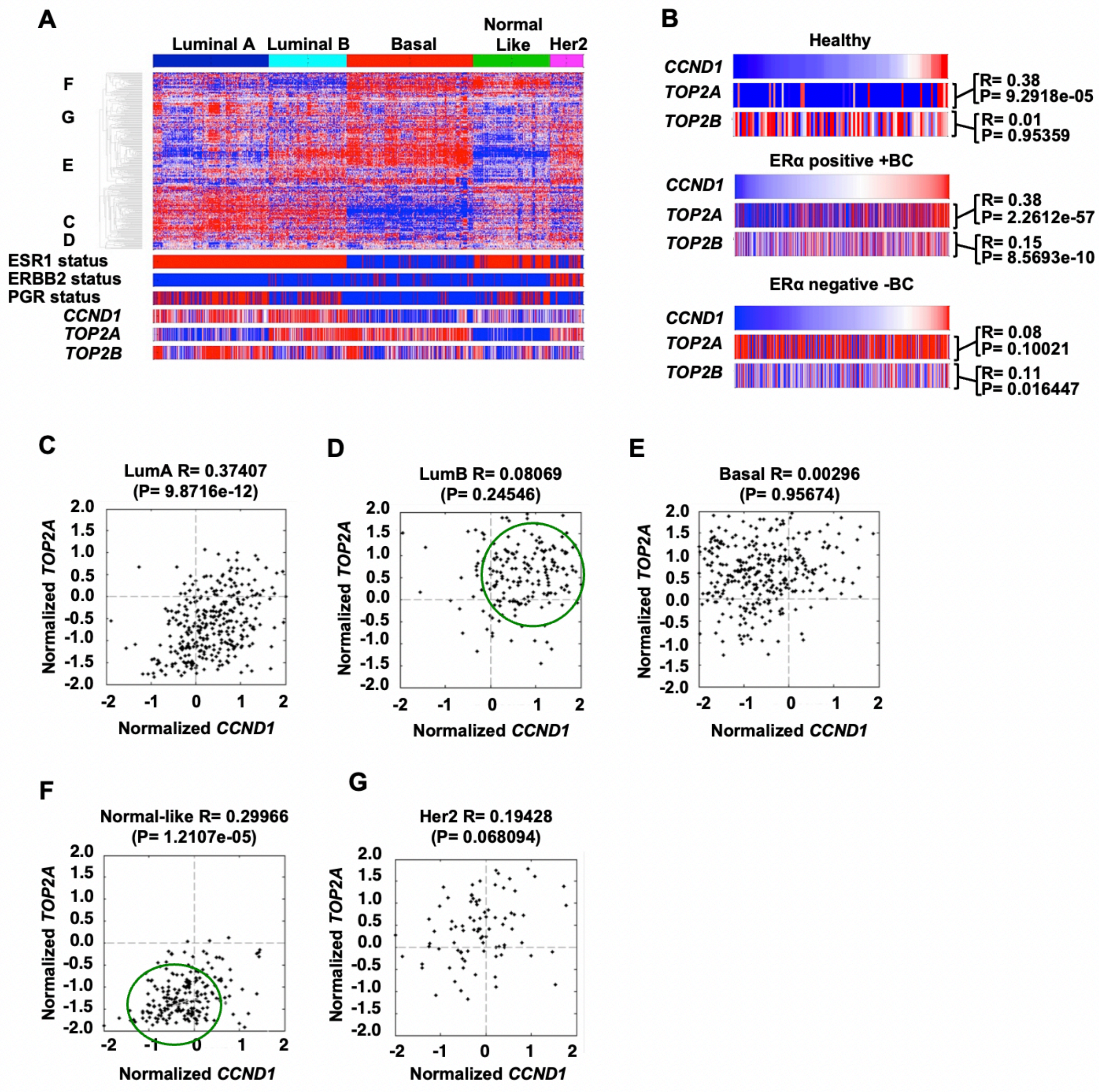

Figure S4. Cyclin D1 correlates with Top2A expression in luminal B and normal like breast cancer subtypes. (A). Heatmap depiction of samples from combined breast cancer microarray datasets that were assigned to the five breast cancer microarray subtypes. The predicted estrogen receptor α (ESR1), epidermal growth factor receptor ERBB2, progesterone receptor (PGR), status are depicted together with TOP2A and TOP2B expression with cyclin D1 (CCND1) expression level across the 5 subtypes. (B). Relative enrichment of TOP2A in $ER\alpha^{+}$ vs. $ER\alpha^{-}$ breast cancer. (C-G). Scatter plots depict CCND1 transcript level versus TOP2A, shows the relationship between TOP2A and cyclin D1 expression in luminal B and normal-like subtype-specific (green circle). The green circle highlights luminal B and normal like subtype in which TOP2A and cyclin D1 expression level show a co-segregation.

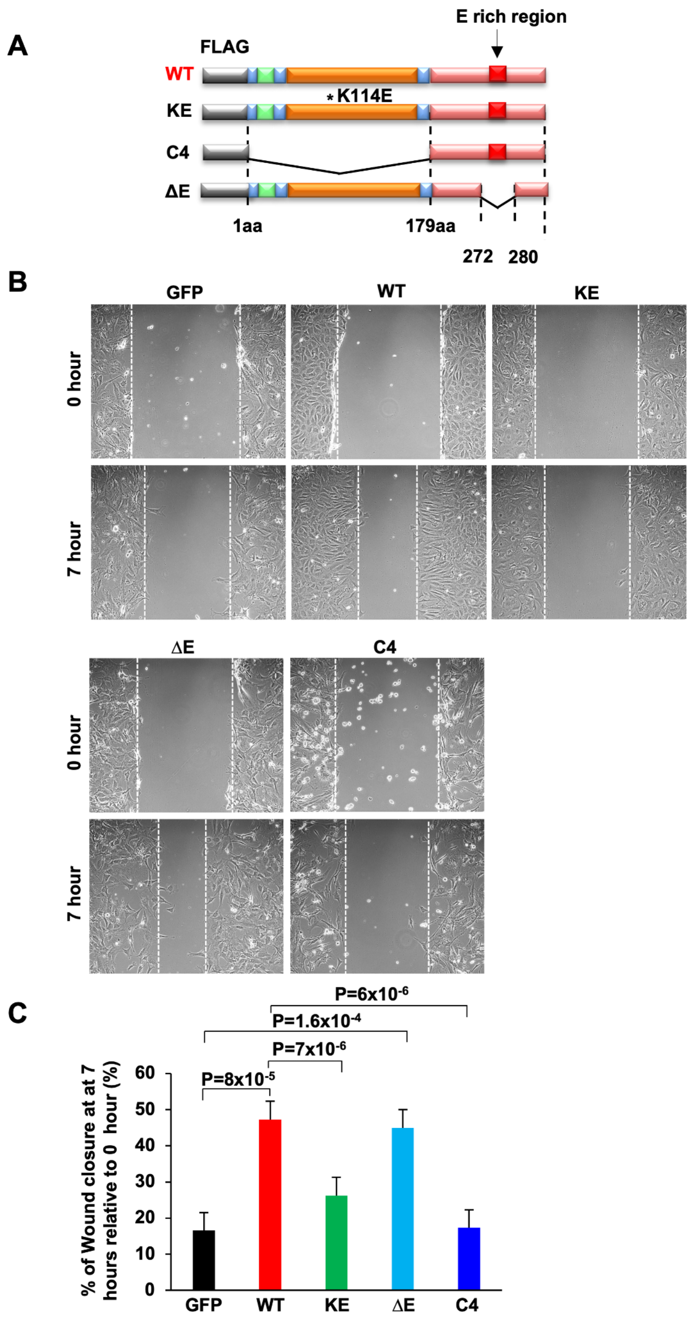

Figure S5. Cyclin D1^{WT} and cyclin D1$^{\Delta E}$ promote cellular migration in wound healing assays. (A). Schematic representation of cyclin D1 expression vectors. (B). Representative wound healing assays performed at 0 hour and 7 hours showing phase contrast microscopy of cyclin D1$^{-/-}$ 3T3 cells, transduced with an expression vector for cyclin D1 (Ccnd1$^{-/-CD1WT}$), cyclin D1^{KE}, cyclin D1$^{\Delta E}$, cyclin D1^{C4} or GFP control. (C). Mean data ± SEM shown as percentage of wound closure at 7 hours compared with 0 hour for each expression plasmid. The pro-migratory phenotype is restored by cyclin D1^{WT} and cyclin D1$^{\Delta E}$ but not by cyclin D1^{C4} or cyclin D1^{KE}.

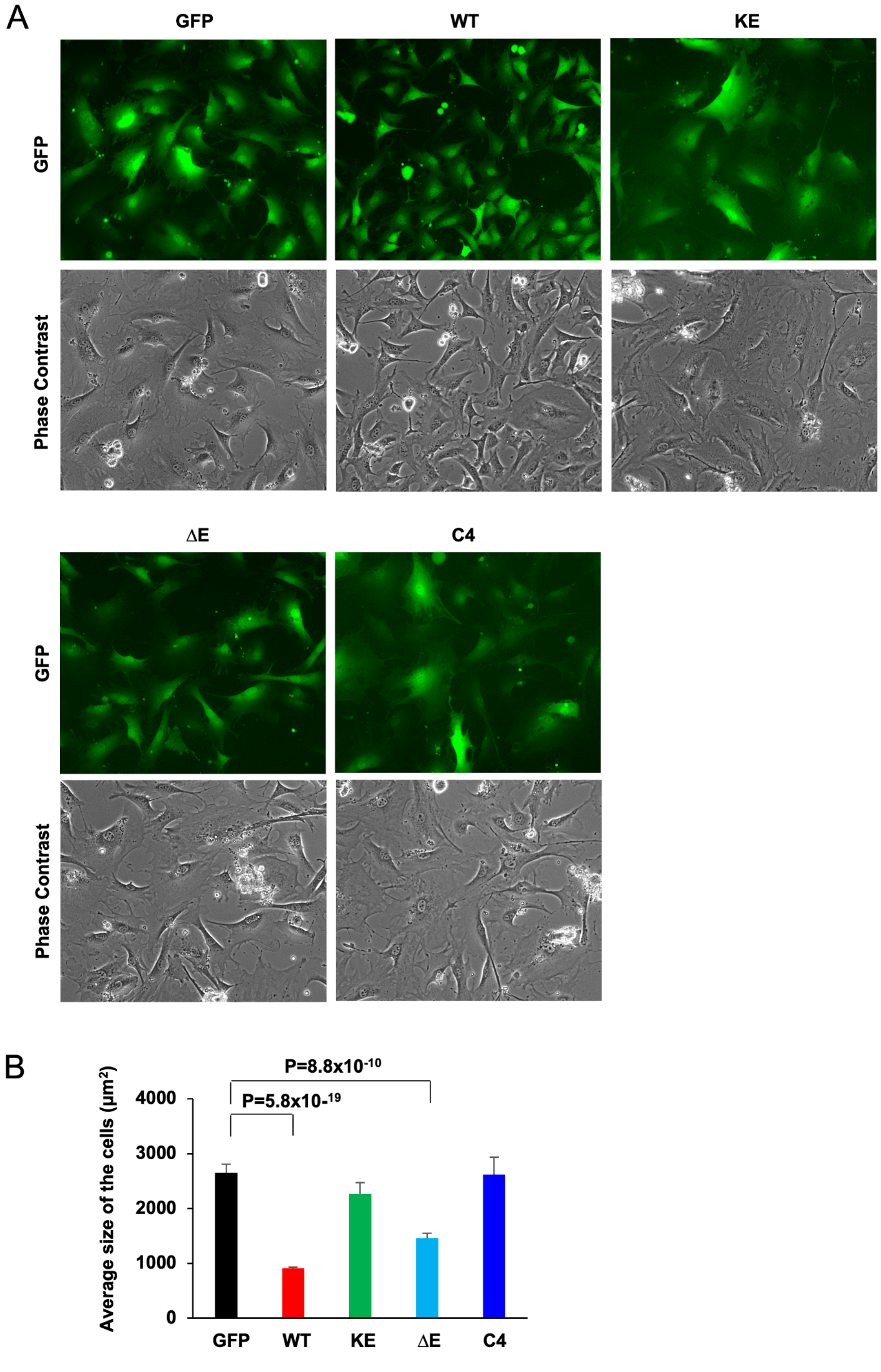

Figure S6. Cyclin D1WT and cyclin D1 E induce cellular polarization. (A). Representative fluorescence and phase contrast microscopy of Cyclin D1$^{-/-}$ 3T3 cells, transduced with an expression vector for either cyclin D1 (Ccnd1$^{-/-}$-Cyclin D1^{WT}), cyclin D1$^{\Delta E}$, cyclin D1^{C4} or GFP control. (B). Mean data ± SEM shown as average size of the cells. The polarized morphology is restored by cyclin D1^{WT} and cyclin D1$^{\Delta E}$ but not by cyclin D1^{KE} or cyclin D1^{C4}.

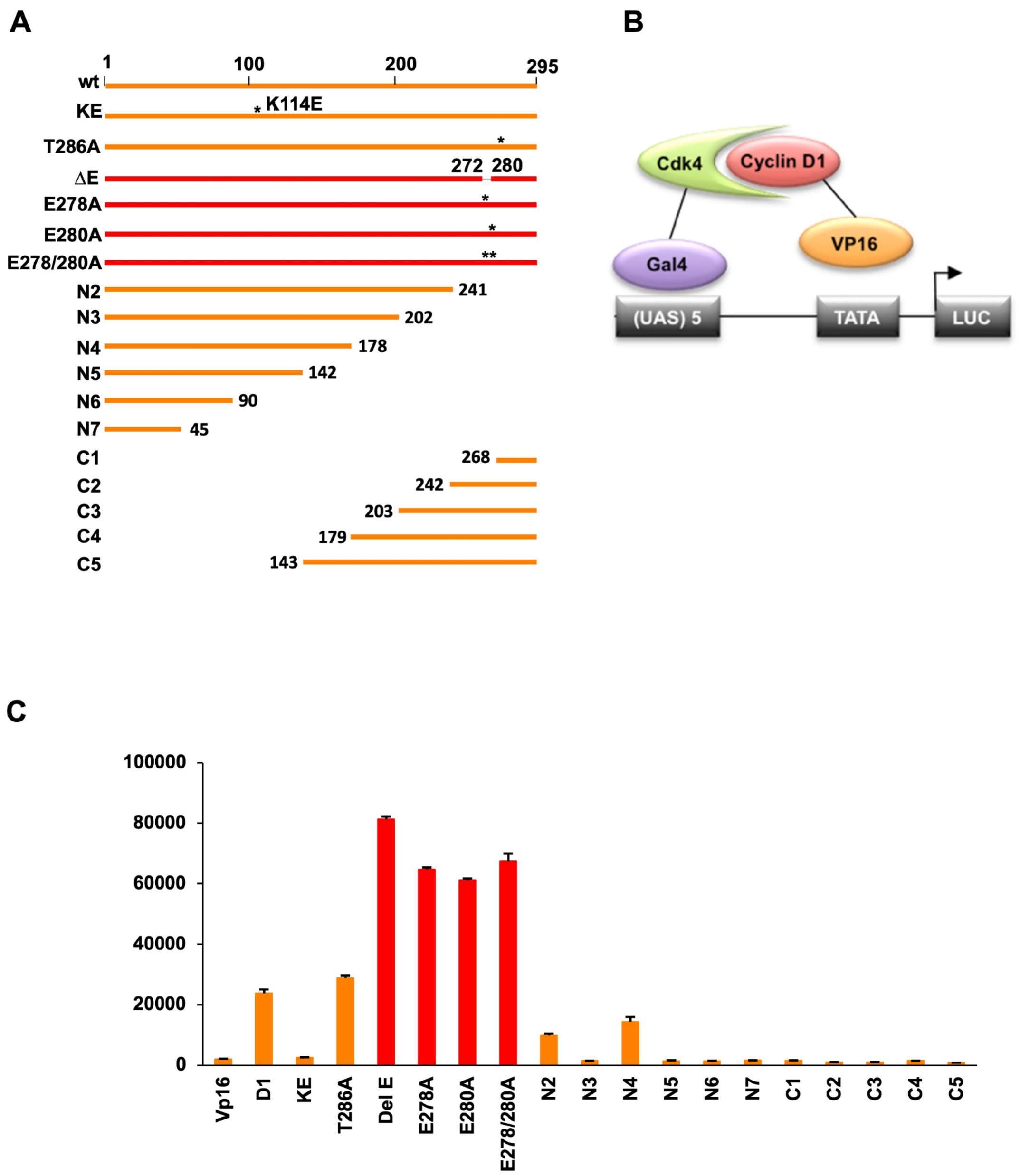

Figure S7. Mammalian 2-hybrid interaction of cyclin D1 with Cdk4. (A). Schematic representation of cyclin D1-VP16 fusion constructs. (B). Schematic representation of Mammalian 2-hybrid vectors. (C). Interaction of cyclin $D1^{WT}$ and cyclin $D1^{\Delta E}$ shown as mean ± SEM for N>5 separate transfections conducted in HEK293T cells.

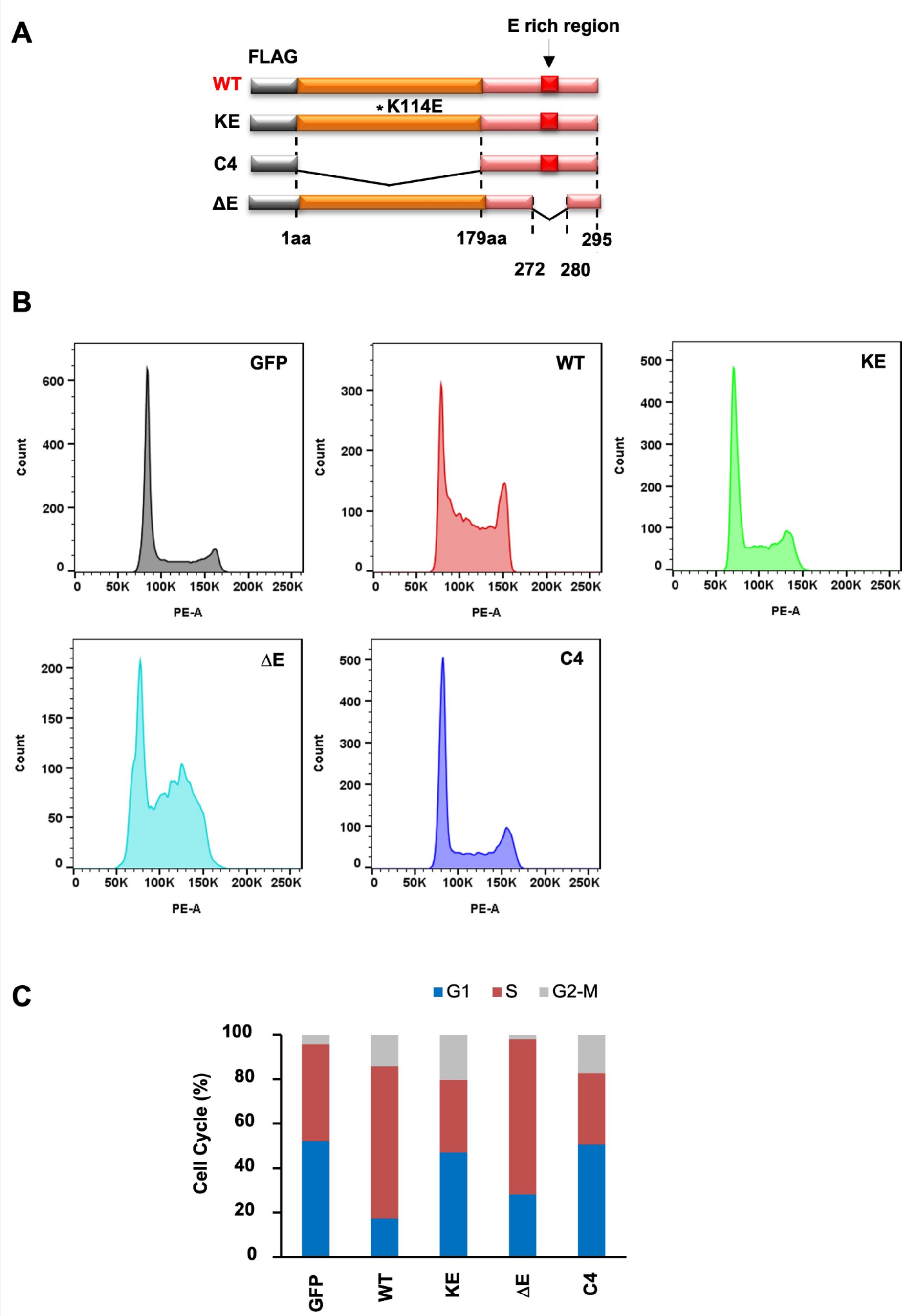

Figure S8. Cell cycle regulation of G2/M by cyclin D1 E region. (A). Schematic representation of cyclin D1 expression vectors used to rescue cyclin D1$^{-/-}$ 3T3 cells. (B). Cell cycle analysis of cyclin D1 mutant rescue cell lines by FACS and the bar graph of the results was showed in (C).

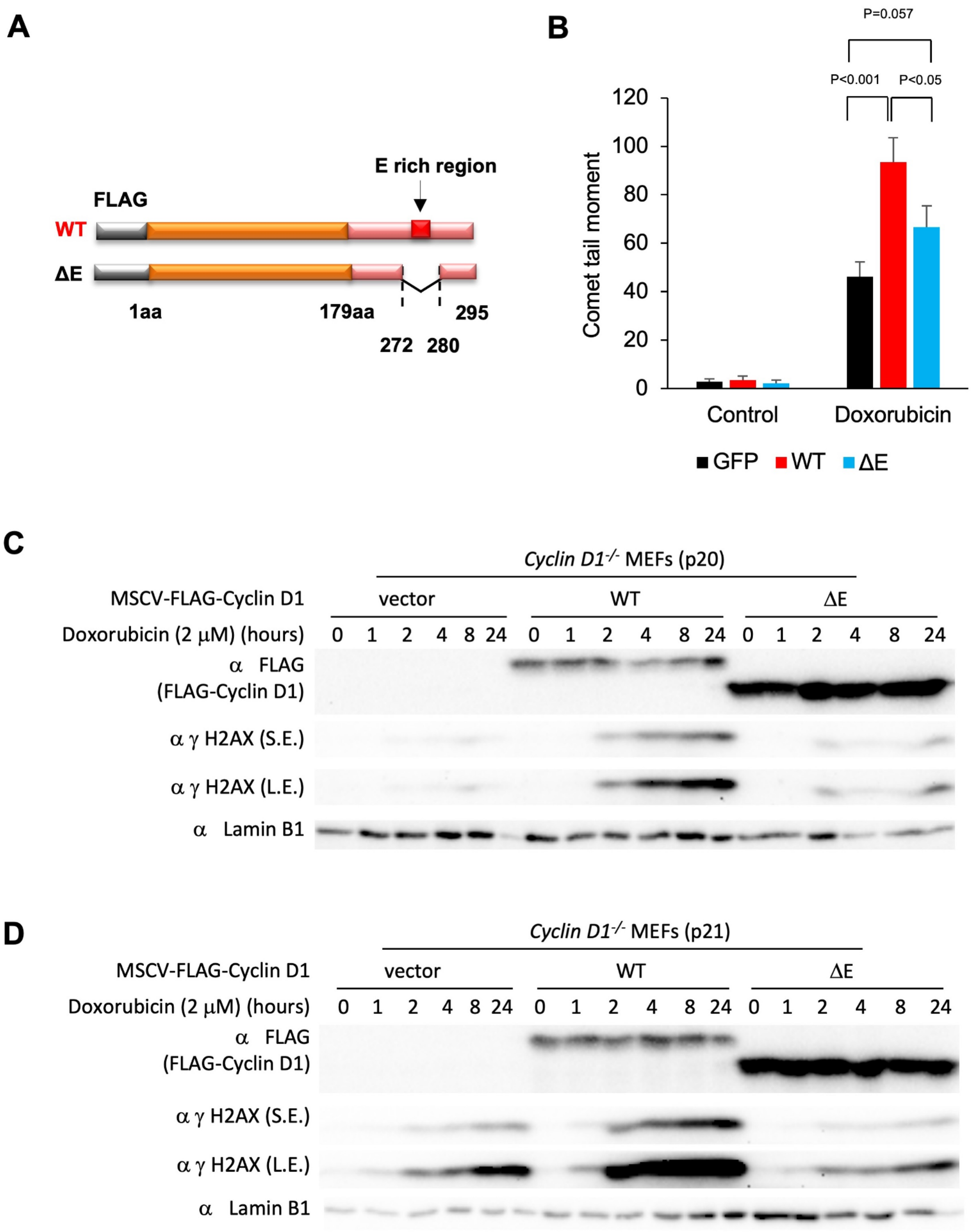

Figure S9. Induction of comet tail formation involves the cyclin D1 E region. (A). Schematic representation of cyclin D1 expression vectors used to rescue *cyclin D1*$^{-/-}$ 3T3 cells. (B). Comet tail moment formation after Doxorubicin treatment. Data are shown for analysis of (GFP control N=83, Dox N=104, Cyclin D1 WT control N=80., Dox N=73., Cyclin D1 ΔE (DelE) control N=87, Dox N=85). P value were determined using the Student t test. (C and D). Western-blot analysis showed increased γ-H2AX abundance in cyclin D1^{WT} rescued *cyclin D1*$^{-/-}$ MEFs at passage 20 (p20) (C) and passage 21 (p21) (D) but not in cyclin D1$^{\Delta E}$ rescue cells upon doxorubicin treatment.

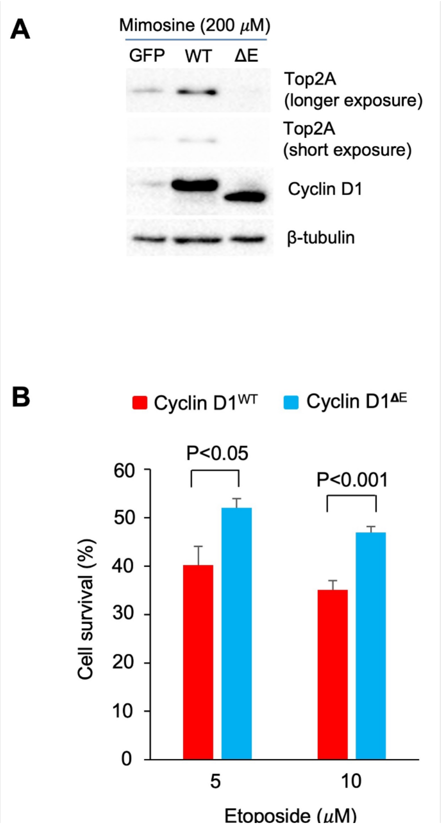

Figure S10. Cyclin D1 E domain contributes to Top2A induction and inhibitors sensitivity. *Cyclin D1$^{-/-}$* 3T3 cells, transduced with either an expression vector for cyclin D1^{WT}, cyclin D1$^{\Delta E}$ or vector control. were assessed for Top2A abundance after treatment with mimosine (200 μM, 24 hrs) to arrest the cells prior to DNA replication. Antibodies used were as shown in the figure. B-tubulin is a protein loading control. (B). Cell viability of the *cyclin D1$^{-/-}$* 3T3 cells, transduced with an expression vector for cyclin D1^{WT} or cyclin D1$^{\Delta E}$ in response to the Top2A inhibitor etoposide (5 μM, 10 μM) for 24 hrs. Data are shown as mean ± SEM compared with vector control established as 100%, with the P value was established by the student t test (N=6).

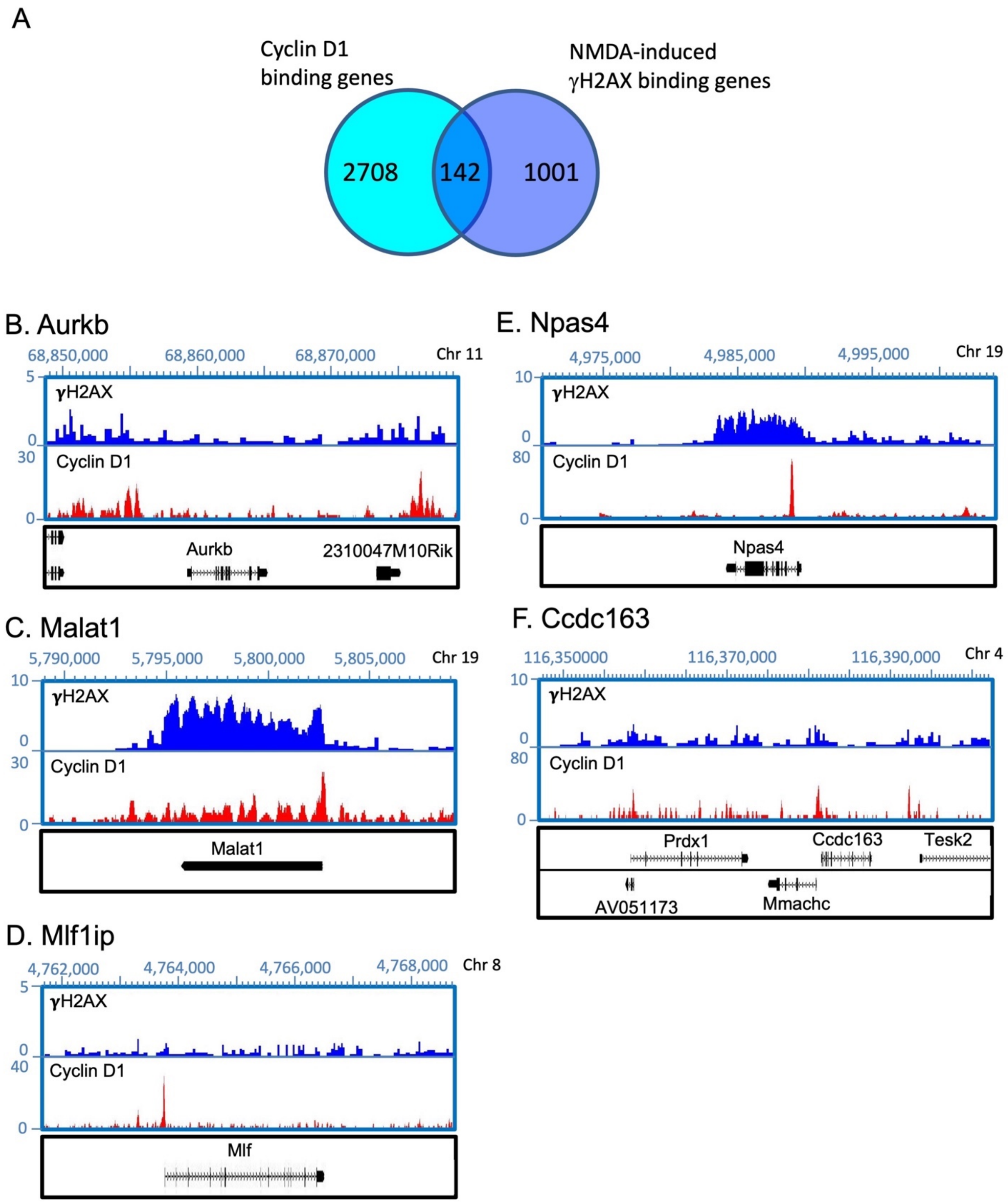

Figure S11. Cyclin D1 and gH2AX colocalize in ChIP-Seq. (A). Venn diagram depicting the number of gene identified in cyclin D1 ChIP seq[1] shows ~ 14% overlap with γH2AX ChIP Seq[2]. (B-F). Representative examples of integrated genome browser visualization of tag density profiles for ChIP-Seq Cyclin D1 WT and γH2AX. Selected genes are: *AurkB, Malat1, Mlf1p*, *Npas4, and Ccdc163*.

SUPPLEMENTAL TABLES

Table S1. Oligonucleotide probes used in ChIP.

Target	Region	Primer	Sequence	Product length
Top2A	Specific 1	Forward	atcaccgactcgctctcatt	188 bp
		Reverse	gcacatggaccttcgtcatt	
	Specific 2	Forward	cgccttttcgaataaagcaa	183 bp
		Reverse	aaggacggcttagtcaacca	
	NS1	Forward	ccttagcctgctgtgtctcc	237 bp
		Reverse	cagtgggacaccaaggttct	
	NS2	Forward	gccctatgctagagggaacc	155 bp
		Reverse	ctcacagggcacagctgata	
Mif1	Specific 1	Forward	ataacgtgtcgcgttcaaca	434 bp
		Reverse	aaaggcagggactccaaact	
	Specific 2	Forward	aggccggggagttctaaat	243 bp
		Reverse	aaaggcagggactccaaact	
	NS1	Forward	gaaatggtagggggaccatc	245 bp
		Reverse	tcacttaaacccgggaagtg	
	NS2	Forward	gcaagtccaagactgggaaa	250 bp
		Reverse	gttgcactgcattcttggtg	
Zw10	Specific 1	Forward	gggagggccataaaggatta	165 bp
		Reverse	atcggatgtgggtgtgaaat	
	Specific 2	Forward	gctgagcccaatgaatgaat	158 bp
		Reverse	gttgggttggtttcgvtaga	
	NS1	Forward	ggagataaggtgctgggtca	240 bp
		Reverse	atcggatgtgggtgtgaaat	
	NS2	Forward	attgatggctccctgtatgg	163 bp
		Reverse	attgggagagggctcttgtt	

Table S2. Proteins containing the cyclin D I intrinsically disordered domains (IDD) E box motif.

Gene Symbol	Description	Code	Reference:
ReACIN1	Apoptotic chromatin condensation inducer in the nucleus isoform 1	NP_055792	[3]
ADRA2B	Adrenergic, alpha-2B	AAY43127	[4]
AEBP1	Adipocyte enhancer binding protein 1 precursor variant	BAD92981	N/A
AEBP2	Zinc finger protein AEBP2 isoform b	NP_001107648	[5]
ALMS1	Alstrom syndrome protein 1	NP_055935	[6]
ANP32E	Acidic leucine-rich nuclear phosphoprotein 32 family member E isoform 1	NP_112182	[7]
ARID4B	ARID4B variant protein	BAE06114	[8]
ATRX	Zinc finger helicase	AAC51655	[9]
BAZ1B	bromodomain adjacent to zinc finger domain 1B	BAA89210	[10]
BCL11A	B-cell lymphoma/leukaemia 11A extra long form	CAC17723	[11]
BCL11B	B-cell lymphoma/leukemia 11B isoform 1	NP_612808	[12]
BCORL1	RecName: Full=BCL-6 corepressor-like protein 1	Q5H9F3	[13]
BZRAP1	benzodiazapine receptor (peripheral) associated protein 1	EAW94468	N/A
C11orf95	uncharacterized protein C11orf95	NP_001138408	N/A
C17orf85	uncharacterized protein C17orf85	NP_001107590	N/A
C9orf174	unnamed protein product	BAG64113	N/A
CACNA1F	calcium channel, voltage-dependent, alpha 1F subunit, isoform CRA_b	EAW50678	N/A
CCDC108	coiled-coil domain-containing protein 108 isoform 1	NP_919278	N/A
CCDC136	KIAA1793 protein	BAB47422	N/A
CCND1	G1/S-specific cyclin-D1	NP_444284	[14]
CCNI	unnamed protein product	BAH13410	N/A
CDK11A	Cyclin-dependent kinase 11A	Q9UQ88	[15]
CDK11B	Cyclin-dependent kinase 11B	P21127	[16]
CDK11B	PITSLRE protein kinase beta SV13 isoform	AAC72090	[16]
CELSR2	cadherin, EGF LAG seven-pass G-type receptor 2	EAW56372	N/A
CENPB	major centromere autoantigen B	NP_001801	[17]
CERS3	LAG1 longevity assurance homolog 3	EAX02267	N/A
CHD3	chromodomain-helicase-DNA-binding protein 3 isoform 3	NP_001005271	[18]
CHGA	chromogranin A variant	BAD97101	N/A
CHIC1	cysteine-rich hydrophobic domain 1 protein	NP_001034929	N/A
CLSPN	claspin isoform 1	NP_071394	[19]
CLSTN1	KIAA0911 protein	BAA74934	N/A
CNGB1	cyclic nucleotide-gated cation channel beta-1 isoform a	NP_001288	N/A
CNKSR2	connector enhancer of kinase suppressor of Ras 2, isoform CRA_a	EAW98976	N/A
CNPY4	protein canopy homolog 4 precursor	NP_689968	N/A
CSRNP3	cysteine/serine-rich nuclear protein 3	NP_079245	N/A
CUL9	novel protein	CAI20204	[20]
DAXX	death-associated protein 6, isoform CRA_b	EAX03724	[21]
DCAF8	DDB1- and CUL4-associated factor 8	NP_056541	N/A
DCAF8L2	DDB1- and CUL4-associated factor 8-like protein 2	NP_001130005	N/A
DHX37	probable ATP-dependent RNA helicase DHX37	NP_116045	N/A
EHMT2	unnamed protein product	BAH13838	[22]

EIF3D	eukaryotic translation initiation factor 3 subunit D	NP_003744	[23]
EIF5B	KIAA0741 protein	BAA34461	N/A
EP400	E1A-binding protein p400	Q96L91	[7]
EPHA6	ephrin type-A receptor 6 isoform a	NP_001073917	N/A
FAM212A	protein FAM212A	NP_976248	N/A
FAM9A	family with sequence similarity 9	EAW98761	N/A
FBLN2	fibulin 2	AAN05435	N/A
FBXO3	unnamed protein product	BAA91991	N/A
FKBP8	hypothetical protein	CAD98028	N/A
FTSJ3	FtsJ homolog 3	AAH36710	[24]
GTF3C3	general transcription factor 3C polypeptide 3 isoform	NP_036218	N/A
GTF3C5	general transcription factor 3C polypeptide 5 isoform 1	NP_001116295	N/A
GZF1	unnamed protein product	BAF82503	[25]
HMGB3	high-mobility group box 3, isoform CRA_b	EAW99398	N/A
HMGB3P30	hCG1644442	EAX02628	N/A
HNRNPU	heterogeneous nuclear ribonucleoprotein U isoform a	NP_114032	[26]
HOMEZ	KIAA1443 protein	BAA92681	N/A
HRC	histidine rich calcium binding protein	ACH88003	N/A
IL27	interleukin-27 subunit alpha precursor	NP_663634	N/A
IRX6	iroquois-class homeodomain protein IRX-6	NP_077311	N/A
ISY1	pre-mRNA-splicing factor ISY1 homolog isoform 1	NP_001186398	[27]
ISY1-RAB43	KIAA1160 protein	BAA86474	N/A
KAT6B	histone acetyltransferase KAT6B isoform 1	NP_036462	N/A
KCNA4	potassium voltage-gated channel subfamily A member 4	NP_002224	N/A
KCTD1	BTB/POZ domain-containing protein KCTD1 isoform b	NP_001136202	[28]
KDM2A	KIAA1004 protein	BAA76848	[29]
KIF1A	KIF1A variant protein	BAG06726	N/A
KIF21A	kinesin family member 21A, isoform CRA_d	EAW57806	N/A
LEPREL2	prolyl 3-hydroxylase 3 precursor	NP_055077	N/A
LPPR3	lipid phosphate phosphatase-related protein type 3 isoform 1	NP_079164	N/A
MAP3K9	mitogen-activated protein kinase kinase kinase 9	NP_149132	N/A
MAPK8IP2	Mitogen-activated protein kinase 8 interacting protein 2	AAH47527	N/A
MICAL3	protein-methionine sulfoxide oxidase MICAL3 isoform 1	NP_056056	N/A
MIER1	unnamed protein product	BAC11339	[30]
MYO15B	KIAA1783 protein	BAB47412	N/A
MYT1	KIAA0835 protein	BAA74858	[31]
MYT1L	myelin transcription factor 1-like	AAF14051	N/A
NACAD	NAC-alpha domain-containing protein 1	NP_001139806	N/A
NADK	NAD kinase isoform 2	NP_001185923	N/A
NAP1L2	nucleosome assembly protein 1-like 2	NP_068798	N/A
NCL	nucleolin	NP_005372	[32]
NEUROD1	BETA2	BAA36519	N/A
NEUROD2	neurogenic differentiation factor 2	NP_006151	N/A
NGEF	Neuronal guanine nucleotide exchange factor	AAH31573	N/A
NOP9	chromosome 14 open reading frame 21, isoform CRA_b	EAW66035	N/A
P4HA2	procollagen-proline, 2-oxoglutarate 4-dioxygenase (proline 4-hydroxylase), alpha polypeptide II, isoform CRA_e	EAW62347	N/A
PAXIP1	PAX-interacting protein 1	NP_031375	[33]
PCGF6	hMBLR	BAB40779	[34]
PELP1	PELP1	AAC17708	[35]
PHF23	NUP98/PHF23 fusion protein	ABK59096	N/A

PIAS4	E3 SUMO-protein ligase PIAS4	NP_056981	[36]
PIK3R5	phosphoinositide 3-kinase regulatory subunit 5 isoform 1	NP_055123	N/A
PLCZ1	phospholipase C, zeta 1, isoform CRA_b	EAW96389	[37]
PLEKHG5	KIAA0720 protein	BAA34440	N/A
PODN	Podocan	AAH30608	N/A
PODXL2	podocalyxin-like protein	AAQ89454	N/A
PPARGC1B	peroxisome proliferative activated receptor, gamma, coactivator 1, beta, isoform CRA_d	EAW61762	N/A
PRDM2	zinc finger protein RIZ	AAC50820	[38]
PRKCSH	unnamed protein product	BAG58917	N/A
PRM3	protamine-3	NP_067070	N/A
PROCA1	RecName: Full=Protein PROCA1	Q8NCQ7	N/A
PRX	periaxin isoform 2	NP_870998	N/A
PVRL1	poliovirus receptor-related 1 alpha isoform	AAR88251	N/A
RANGAP1	Ran GTPase activating protein 1, isoform CRA_a	EAW60420	[39]
RBM19	KIAA0682 protein	BAA31657	N/A
RGL2	ral guanine nucleotide dissociation stimulator-like 2 isoform 1	NP_004752	N/A
RRP12	RRP12-like protein isoform 1	NP_055994	N/A
RTN4	KIAA0886 protein	BAA74909	N/A
SCAF1	FLJ00034 protein	BAB15734	N/A
SCRIB	scribbled homolog (Drosophila), isoform CRA_b	EAW82197	N/A
SENP3	SUMO1/sentrin/SMT3 specific peptidase 3, isoform CRA_a	EAW90170	[36]
SETD1B	histone-lysine N-methyltransferase	AEG67286	[40]
SHROOM4	KIAA1202 protein	BAA86516	N/A
SKIDA1	SKI/DACH domain-containing protein 1	NP_997254	N/A
SLC24A1	sodium/potassium/calcium exchanger 1 isoform 1	NP_004718	N/A
SLC38A5	solute carrier family 38, member 5, isoform CRA_b	EAW50786	N/A
SLC4A1AP	unnamed protein product	BAA91718	N/A
SLC4A3	anion exchanger SLC4A3	AAN34939	N/A
SMARCA4	SMARCA4 protein	AAI36645	[41]
SMYD5	SET and MYND domain-containing protein 5	NP_006053	N/A
SNAPC5	snRNA-activating protein complex subunit 5	NP_006040	N/A
SPRYD3	SPRY domain containing 3, isoform CRA_a	EAW96661	N/A
STAC3	SH3 and cysteine-rich domain-containing protein 3	NP_659501	N/A
SUPT5H	suppressor of Ty 5 homolog variant	BAD92494	[42]
TAOK2	TAOK2 protein, partial	AAH51798	[43]
TEX2	testis-expressed sequence 2 protein	NP_060939	N/A
TIMELESS	timeless homolog (Drosophila)	EAW96939	[44]
TMEM132A	transmembrane protein 132A, isoform CRA_d	EAW73911	N/A
TPRN	taperin >sp|Q4KMQ1.2|TPRN_HUMAN RecName: Full=Taperin	NP_001121700	N/A
TRAK1	unnamed protein product	BAH12083	N/A
TRIM26	tripartite motif-containing protein 26	NP_003440	N/A
TTBK1	tau tubulin kinase 1, isoform CRA_c	EAX04169	N/A
UQCRH	unnamed protein product	CAA68733	N/A
UQCRHL	ubiquinol-cytochrome c reductase hinge protein-like	NP_001083060	N/A
VSIG10	V-set and immunoglobulin domain-containing protein 10 precursor	NP_061959	N/A
WDR70	hypothetical protein	CAC21644	N/A
WEE1	WEE1 homolog (S. pombe)	AAH51831	N/A
WIZ	Protein Wiz	O95785	N/A
YTHDC1	YTH domain-containing protein 1 isoform 1	NP_001026902	N/A

ZBTB47	zinc finger and BTB domain-containing protein 47	NP_660149	N/A
ZBTB7C	zinc finger and BTB domain-containing protein 7C	NP_001034449	[45]
ZEB1	zinc finger E-box-binding homeobox 1 isoform f	NP_001167567	[46]
ZFHX3	zinc finger homeobox protein 3 isoform A	NP_008816	[47]
ZFP91	E3 ubiquitin-protein ligase ZFP91 isoform 1	NP_444251	N/A
ZFP91-CNTF	testis specific ZFP91	BAB63374	N/A
ZNF428	Zinc finger protein 428	AAH45799	N/A
ZNF830	coiled-coil domain containing 16	EAW80200	N/A

Table S3. Active Motif MODified™ Histone Peptide Array* (Catalog Nos. 13001 & 13005)

Modification number	*Peptide location*	*Peptide sequence*	*name*	*Mod1*	*Mod2*	*Mod 3*	*Mod 4*	*N-terminus*
1	A 1	A R T K Q T A R K S T G G K A P R K Q	H3 1-19	unmod				free
2	A 2	A Rme2s T K Q T A R K S T G G K A P R K Q	H3 1-19	R2me2s				free
3	A 3	A Rme2a T K Q T A R K S T G G K A P R K Q	H3 1-19	R2me2a				free
4	A 4	A Cit T K Q T A R K S T G G K A P R K Q	H3 1-19	R2Citr				free
5	A 5	A R pT K Q T A R K S T G G K A P R K Q	H3 1-19	T3P				free
6	A 6	A R T Kme1 Q T A R K S T G G K A P R K Q	H3 1-19	K4me1				free
7	A 7	A R T Kme2 Q T A R K S T G G K A P R K Q	H3 1-19	K4me2				free
8	A 8	A R T Kme3 Q T A R K S T G G K A P R K Q	H3 1-19	K4me3				free
9	A 9	A R T Kac Q T A R K S T G G K A P R K Q	H3 1-19	K4ac				free
10	A10	A R T K Q T A Rme2s K S T G G K A P R K Q	H3 1-19	R8me2s				free
11	A11	A R T K Q T A Rme2a K S T G G K A P R K Q	H3 1-19	R8me2a				free
12	A12	A R T K Q T A Cit K S T G G K A P R K Q	H3 1-19	R8Citr				free
13	A13	A R T K Q T A R Kme1 S T G G K A P R K Q	H3 1-19	K9me1				free
14	A14	A R T K Q T A R Kme2 S T G G K A P R K Q	H3 1-19	K9me2				free
15	A15	A R T K Q T A R Kme3 S T G G K A P R K Q	H3 1-19	K9me3				free
16	A16	A R T K Q T A R Kac S T G G K A P R K Q	H3 1-19	K9ac				free
17	A17	A R T K Q T A R K pS T G G K A P R K Q	H3 1-19	S10P				free
18	A18	A R T K Q T A R K S pT G G K A P R K Q	H3 1-19	T11P				free
19	A19	A R T K Q T A R K S T G G Kac A P R K Q	H3 1-19	K14ac				free
20	A20	A Rme2s pT K Q T A R K S T G G K A P R K Q	H3 1-19	R2me2s	T3P			free
21	A21	A Rme2s T Kme1 Q T A R K S T G G K A P R K Q	H3 1-19	R2me2s	K4me1			free
22	A22	A Rme2s T Kme2 Q T A R K S T G G K A P R K Q	H3 1-19	R2me2s	K4me2			free
23	A23	A Rme2s T Kme3 Q T A R K S T G G K A P R K Q	H3 1-19	R2me2s	K4me3			free
24	A24	A Rme2s T Kac Q T A R K S T G G K A P R K Q	H3 1-19	R2me2s	K4ac			free
25	B 1	A Rme2a pT K Q T A R K S T G G K A P R K Q	H3 1-19	R2me2a	T3P			free
26	B 2	A Rme2a T Kme1 Q T A R K S T G G K A P R K Q	H3 1-19	R2me2a	K4me1			free
27	B 3	A Rme2a T Kme2 Q T A R K S T G G K A P R K Q	H3 1-19	R2me2a	K4me2			free
28	B 4	A Rme2a T Kme3 Q T A R K S T G G K A P R K Q	H3 1-19	R2me2a	K4me3			free
29	B 5	A Rme2a T Kac Q T A R K S T G G K A P R K Q	H3 1-19	R2me2a	K4ac			free
30	B 6	A Cit pT K Q T A R K S T G G K A P R K Q	H3 1-19	R2Citr	T3P			free
31	B 7	A Cit T Kme1 Q T A R K S T G G K A P R K Q	H3 1-19	R2Citr	K4me1			free
32	B 8	A Cit T Kme2 Q T A R K S T G G K A P R K Q	H3 1-19	R2Citr	K4me2			free
33	B 9	A Cit T Kme3 Q T A R K S T G G K A P R K Q	H3 1-19	R2Citr	K4me3			free
34	B10	A Cit T Kac Q T A R K S T G G K A P R K Q	H3 1-19	R2Citr	K4ac			free
35	B11	A R pT Kme1 Q T A R K S T G G K A P R K Q	H3 1-19	T3P	K4me1			free
36	B12	A R pT Kme2 Q T A R K S T G G K A P R K Q	H3 1-19	T3P	K4me2			free
37	B13	A R pT Kme3 Q T A R K S T G G K A P R K Q	H3 1-19	T3P	K4me3			free
38	B14	A R pT Kac Q T A R K S T G G K A P R K Q	H3 1-19	T3P	K4ac			free
39	B15	A Rme2s pT Kme1 Q T A R K S T G G K A P R K Q	H3 1-19	R2me2s	T3P	K4me1		free
40	B16	A Rme2s pT Kme2 Q T A R K S T G G K A P R K Q	H3 1-19	R2me2s	T3P	K4me2		free
41	B17	A Rme2s pT Kme3 Q T A R K S T G G K A P R K Q	H3 1-19	R2me2s	T3P	K4me3		free
42	B18	A Rme2s pT Kac Q T A R K S T G G K A P R K Q	H3 1-19	R2me2s	T3P	K4ac		free
43	B19	A Rme2a pT Kme1 Q T A R K S T G G K A P R K Q	H3 1-19	R2me2a	T3P	K4me1		free
44	B20	A Rme2a pT Kme2 Q T A R K S T G G K A P R K Q	H3 1-19	R2me2a	T3P	K4me2		free
45	B21	A Rme2a pT Kme3 Q T A R K S T G G K A P R K Q	H3 1-19	R2me2a	T3P	K4me3		free
46	B22	A Rme2a pT Kac Q T A R K S T G G K A P R K Q	H3 1-19	R2me2a	T3P	K4ac		free
47	B23	A R T K Q T A Rme2a Kme1 S T G G K A P R K Q	H3 1-19	R8me2s	K9me1			free
48	B24	A R T K Q T A Rme2a Kme2 S T G G K A P R K Q	H3 1-19	R8me2s	K9me2			free
49	C 1	A R T K Q T A Rme2a Kme3 S T G G K A P R K Q	H3 1-19	R8me2s	K9me3			free
50	C 2	A R T K Q T A Rme2a Kac S T G G K A P R K Q	H3 1-19	R8me2s	K9ac			free
51	C 3	A R T K Q T A Rme2a K pS T G G K A P R K Q	H3 1-19	R8me2s	S10P			free
52	C 4	A R T K Q T A Rme2a K S pT G G K A P R K Q	H3 1-19	R8me2s	T11P			free
53	C 5	A R T K Q T A Rme2a Kme1 S T G G K A P R K Q	H3 1-19	R8me2a	K9me1			free

54	C 6	A R T K Q T A Rme2a Kme2 S T G G K A P R K Q	H3 1-19	R8me2a	K9me2			free
55	C 7	A R T K Q T A Rme2a Kme3 S T G G K A P R K Q	H3 1-19	R8me2a	K9me3			free
56	C 8	A R T K Q T A Rme2a Kac S T G G K A P R K Q	H3 1-19	R8me2a	K9ac			free
57	C 9	A R T K Q T A Rme2a K pS T G G K A P R K Q	H3 1-19	R8me2a	S10P			free
58	C10	A R T K Q T A Rme2a K S pT G G K A P R K Q	H3 1-19	R8me2a	T11P			free
59	C11	A R T K Q T A Cit Kme1 S T G G K A P R K Q	H3 1-19	R8Citr	K9me1			free
60	C12	A R T K Q T A Cit Kme2 S T G G K A P R K Q	H3 1-19	R8Citr	K9me2			free
61	C13	A R T K Q T A Cit Kme3 S T G G K A P R K Q	H3 1-19	R8Citr	K9me3			free
62	C14	A R T K Q T A Cit Kac S T G G K A P R K Q	H3 1-19	R8Citr	K9ac			free
63	C15	A R T K Q T A Cit K pS T G G K A P R K Q	H3 1-19	R8Citr	S10P			free
64	C16	A R T K Q T A Cit K S pT G G K A P R K Q	H3 1-19	R8Citr	T11P			free
65	C17	A R T K Q T A R Kme1 pS T G G K A P R K Q	H3 1-19	K9me1	S10P			free
66	C18	A R T K Q T A R Kme1 S pT G G K A P R K Q	H3 1-19	K9me1	T11P			free
67	C19	A R T K Q T A R Kme1 S T G G Kac A P R K Q	H3 1-19	K9me1	K14ac			free
68	C20	A R T K Q T A R Kme2 pS T G G K A P R K Q	H3 1-19	K9me2	S10P			free
69	C21	A R T K Q T A R Kme2 S pT G G K A P R K Q	H3 1-19	K9me2	T11P			free
70	C22	A R T K Q T A R Kme2 S T G G Kac A P R K Q	H3 1-19	K9me2	K14ac			free
71	C23	A R T K Q T A R Kme3 pS T G G K A P R K Q	H3 1-19	K9me3	S10P			free
72	C24	A R T K Q T A R Kme3 S pT G G K A P R K Q	H3 1-19	K9me3	T11P			free
73	D 1	A R T K Q T A R Kme3 S T G G Kac A P R K Q	H3 1-19	K9me3	K14ac			free
74	D 2	A R T K Q T A R Kac pS T G G K A P R K Q	H3 1-19	K9ac	S10P			free
75	D 3	A R T K Q T A R Kac S pT G G K A P R K Q	H3 1-19	K9ac	T11P			free
76	D 4	A R T K Q T A R Kac S T G G Kac A P R K Q	H3 1-19	K9ac	K14ac			free
77	D 5	A R T K Q T A R K pS pT G G K A P R K Q	H3 1-19	S10P	T11P			free
78	D 6	A R T K Q T A R K pS T G G Kac A P R K Q	H3 1-19	S10P	K14ac			free
79	D 7	A R T K Q T A R K S pT G G Kac A P R K Q	H3 1-19	T11P	K14ac			free
80	D 8	A R T K Q T A Rme2s Kme1 pS T G G K A P R K Q	H3 1-19	R8me2s	K9me1	S10P		free
81	D 9	A R T K Q T A Rme2s Kme2 pS T G G K A P R K Q	H3 1-19	R8me2s	K9me2	S10P		free
82	D10	A R T K Q T A Rme2s Kme3 pS T G G K A P R K Q	H3 1-19	R8me2s	K9me3	S10P		free
83	D11	A R T K Q T A Rme2s Kac pS T G G K A P R K Q	H3 1-19	R8me2s	K9ac	S10P		free
84	D12	A R T K Q T A Rme2s Kme1 S pT G G K A P R K Q	H3 1-19	R8me2s	K9me1	T11P		free
85	D13	A R T K Q T A Rme2s Kme2 S pT G G K A P R K Q	H3 1-19	R8me2s	K9me2	T11P		free
86	D14	A R T K Q T A Rme2s Kme3 S pT G G K A P R K Q	H3 1-19	R8me2s	K9me3	T11P		free
87	D15	A R T K Q T A Rme2s Kac S pT G G K A P R K Q	H3 1-19	R8me2s	K9ac	T11P		free
88	D16	A R T K Q T A Rme2a Kme1 pS T G G K A P R K Q	H3 1-19	R8me2a	K9me1	S10P		free
89	D17	A R T K Q T A Rme2a Kme2 pS T G G K A P R K Q	H3 1-19	R8me2a	K9me2	S10P		free
90	D18	A R T K Q T A Rme2a Kme3 pS T G G K A P R K Q	H3 1-19	R8me2a	K9me3	S10P		free
91	D19	A R T K Q T A Rme2a Kac pS T G G K A P R K Q	H3 1-19	R8me2a	K9ac	S10P		free
92	D20	A R T K Q T A Rme2a Kme1 S pT G G K A P R K Q	H3 1-19	R8me2a	K9me1	T11P		free
93	D21	A R T K Q T A Rme2a Kme2 S pT G G K A P R K Q	H3 1-19	R8me2a	K9me2	T11P		free
94	D22	A R T K Q T A Rme2a Kme3 S pT G G K A P R K Q	H3 1-19	R8me2a	K9me3	T11P		free
95	D23	A R T K Q T A Rme2a Kac S pT G G K A P R K Q	H3 1-19	R8me2a	K9ac	T11P		free
96	D24	A R T K Q T A Rme2a Kme1 pS pT G G K A P R K Q	H3 1-19	R8me2a	K9me1	S10P	T11P	free
97	E 1	A R T K Q T A Rme2a Kme2 pS pT G G K A P R K Q	H3 1-19	R8me2a	K9me2	S10P	T11P	free
98	E 2	A R T K Q T A Rme2a Kme3 pS pT G G K A P R K Q	H3 1-19	R8me2a	K9me3	S10P	T11P	free
99	E 3	A R T K Q T A Rme2a Kac pS pT G G K A P R K Q	H3 1-19	R8me2a	K9ac	S10P	T11P	free
100	E 4	A Rme2s T Kme1 Q T A Rme2s K S T G G K A P R K Q	H3 1-19	R2me2s	K4me1	R8me2s		free
101	E 5	A Rme2s T Kme2 Q T A Rme2s K S T G G K A P R K Q	H3 1-19	R2me2s	K4me2	R8me2s		free
102	E 6	A Rme2s T Kme3 Q T A Rme2s K S T G G K A P R K Q	H3 1-19	R2me2s	K4me3	R8me2s		free
103	E 7	A Rme2s T Kac Q T A Rme2s K S T G G K A P R K Q	H3 1-19	R2me2s	K4ac	R8me2s		free
104	E 8	A Rme2a T Kme1 Q T A Rme2a K S T G G K A P R K Q	H3 1-19	R2me2a	K4me1	R8me2a		free
105	E 9	A Rme2a T Kme2 Q T A Rme2a K S T G G K A P R K Q	H3 1-19	R2me2a	K4me2	R8me2a		free
106	E10	A Rme2a T Kme3 Q T A Rme2a K S T G G K A P R K Q	H3 1-19	R2me2a	K4me3	R8me2a		free
107	E11	A Rme2a T Kac Q T A Rme2a K S T G G K A P R K Q	H3 1-19	R2me2a	K4ac	R8me2a		free
108	E12	A Rme2s T Kme1 Q T A R Kme1 S T G G K A P R K Q	H3 1-19	R2me2s	K4me1	K9me1		free
109	E13	A Rme2s T Kme2 Q T A R Kme1 S T G G K A P R K Q	H3 1-19	R2me2s	K4me2	K9me1		free
110	E14	A Rme2s T Kme3 Q T A R Kme1 S T G G K A P R K Q	H3 1-19	R2me2s	K4me3	K9me1		free

111	E15	A Rme2s T Kac Q T A R Kme1 S T G G K A P R K Q	H3 1-19	R2me2s	K4ac	K9me1		free
112	E16	A Rme2a T Kme1 Q T A R Kme2 S T G G K A P R K Q	H3 1-19	R2me2a	K4me1	K9me2		free
113	E17	A Rme2a T Kme2 Q T A R Kme2 S T G G K A P R K Q	H3 1-19	R2me2a	K4me2	K9me2		free
114	E18	A Rme2a T Kme3 Q T A R Kme2 S T G G K A P R K Q	H3 1-19	R2me2a	K4me3	K9me2		free
115	E19	A Rme2a T Kac Q T A R Kme2 S T G G K A P R K Q	H3 1-19	R2me2a	K4ac	K9me2		free
116	E20	A Rme2s T Kme1 Q T A R Kme3 S T G G K A P R K Q	H3 1-19	R2me2s	K4me1	K9me3		free
117	E21	A Rme2s T Kme2 Q T A R Kme3 S T G G K A P R K Q	H3 1-19	R2me2s	K4me2	K9me3		free
118	E22	A Rme2s T Kme3 Q T A R Kme3 S T G G K A P R K Q	H3 1-19	R2me2s	K4me3	K9me3		free
119	E23	A Rme2s T Kac Q T A R Kme3 S T G G K A P R K Q	H3 1-19	R2me2s	K4ac	K9me3		free
120	E24	A Rme2a T Kme1 Q T A R Kac S T G G K A P R K Q	H3 1-19	R2me2a	K4me1	K9ac		free
121	F 1	A Rme2a T Kme2 Q T A R Kac S T G G K A P R K Q	H3 1-19	R2me2a	K4me2	K9ac		free
122	F 2	A Rme2a T Kme3 Q T A R Kac S T G G K A P R K Q	H3 1-19	R2me2a	K4me3	K9ac		free
123	F 3	A Rme2a T Kac Q T A R Kac S T G G K A P R K Q	H3 1-19	R2me2a	K4ac	K9ac		free
124	F 4	A R T Kme1 Q T A Rme2s Kme1 S T G G K A P R K Q	H3 1-19	K4me1	R8me2s	K9me1		free
125	F 5	A R T Kme2 Q T A Rme2s Kme1 S T G G K A P R K Q	H3 1-19	K4me2	R8me2s	K9me1		free
126	F 6	A R T Kme3 Q T A Rme2s Kme1 S T G G K A P R K Q	H3 1-19	K4me3	R8me2s	K9me1		free
127	F 7	A R T Kac Q T A Rme2s Kme1 S T G G K A P R K Q	H3 1-19	K4ac	R8me2s	K9me1		free
128	F 8	A R T Kme1 Q T A Rme2a Kme1 S T G G K A P R K Q	H3 1-19	K4me1	R8me2a	K9me1		free
129	F 9	A R T Kme2 Q T A Rme2a Kme1 S T G G K A P R K Q	H3 1-19	K4me2	R8me2a	K9me1		free
130	F10	A R T Kme3 Q T A Rme2a Kme1 S T G G K A P R K Q	H3 1-19	K4me3	R8me2a	K9me1		free
131	F11	A R T Kac Q T A Rme2a Kme1 S T G G K A P R K Q	H3 1-19	K4ac	R8me2a	K9me1		free
132	F12	A R T Kme1 Q T A Rme2s Kme2 S T G G K A P R K Q	H3 1-19	K4me1	R8me2s	K9me2		free
133	F13	A R T Kme2 Q T A Rme2s Kme2 S T G G K A P R K Q	H3 1-19	K4me2	R8me2s	K9me2		free
134	F14	A R T Kme3 Q T A Rme2s Kme2 S T G G K A P R K Q	H3 1-19	K4me3	R8me2s	K9me2		free
135	F15	A R T Kac Q T A Rme2s Kme2 S T G G K A P R K Q	H3 1-19	K4ac	R8me2s	K9me2		free
136	F16	A R T Kme1 Q T A Rme2a Kme2 S T G G K A P R K Q	H3 1-19	K4me1	R8me2a	K9me2		free
137	F17	A R T Kme2 Q T A Rme2a Kme2 S T G G K A P R K Q	H3 1-19	K4me2	R8me2a	K9me2		free
138	F18	A R T Kme3 Q T A Rme2a Kme2 S T G G K A P R K Q	H3 1-19	K4me3	R8me2a	K9me2		free
139	F19	A R T Kac Q T A Rme2a Kme2 S T G G K A P R K Q	H3 1-19	K4ac	R8me2a	K9me2		free
140	F20	A R T Kme1 Q T A Rme2s Kme3 S T G G K A P R K Q	H3 1-19	K4me1	R8me2s	K9me3		free
141	F21	A R T Kme2 Q T A Rme2s Kme3 S T G G K A P R K Q	H3 1-19	K4me2	R8me2s	K9me3		free
142	F22	A R T Kme3 Q T A Rme2s Kme3 S T G G K A P R K Q	H3 1-19	K4me3	R8me2s	K9me3		free
143	F23	A R T Kac Q T A Rme2s Kme3 S T G G K A P R K Q	H3 1-19	K4ac	R8me2s	K9me3		free
144	F24	A R T Kme1 Q T A Rme2a Kme3 S T G G K A P R K Q	H3 1-19	K4me1	R8me2a	K9me3		free
145	G 1	A R T Kme2 Q T A Rme2a Kme3 S T G G K A P R K Q	H3 1-19	K4me2	R8me2a	K9me3		free
146	G 2	A R T Kme3 Q T A Rme2a Kme3 S T G G K A P R K Q	H3 1-19	K4me3	R8me2a	K9me3		free
147	G 3	A R T Kac Q T A Rme2a Kme3 S T G G K A P R K Q	H3 1-19	K4ac	R8me2a	K9me3		free
148	G 4	A R T Kme1 Q T A Rme2s Kac S T G G K A P R K Q	H3 1-19	K4me1	R8me2s	K9ac		free
149	G 5	A R T Kme2 Q T A Rme2s Kac S T G G K A P R K Q	H3 1-19	K4me2	R8me2s	K9ac		free
150	G 6	A R T Kme3 Q T A Rme2s Kac S T G G K A P R K Q	H3 1-19	K4me3	R8me2s	K9ac		free
151	G 7	A R T Kac Q T A Rme2s Kac S T G G K A P R K Q	H3 1-19	K4ac	R8me2s	K9ac		free
152	G 8	A R T Kme1 Q T A Rme2a Kac S T G G K A P R K Q	H3 1-19	K4me1	R8me2a	K9ac		free
153	G 9	A R T Kme2 Q T A Rme2a Kac S T G G K A P R K Q	H3 1-19	K4me2	R8me2a	K9ac		free
154	G10	A R T Kme3 Q T A Rme2a Kac S T G G K A P R K Q	H3 1-19	K4me3	R8me2a	K9ac		free
155	G11	A R T Kac Q T A Rme2a Kac S T G G K A P R K Q	H3 1-19	K4ac	R8me2a	K9ac		free
156	G12	A Rme2s T Kme1 Q T A Rme2s Kme1 S T G G K A P R K Q	H3 1-19	R2me2s	K4me1	R8me2s	K9me1	free
157	G13	A Rme2s T Kme2 Q T A Rme2s Kme1 S T G G K A P R K Q	H3 1-19	R2me2s	K4me2	R8me2s	K9me1	free
158	G14	A Rme2s T Kme3 Q T A Rme2s Kme1 S T G G K A P R K Q	H3 1-19	R2me2s	K4me3	R8me2s	K9me1	free
159	G15	A Rme2s T Kac Q T A Rme2s Kme1 S T G G K A P R K Q	H3 1-19	R2me2s	K4ac	R8me2s	K9me1	free
160	G16	A Rme2a T Kme1 Q T A Rme2s Kme1 S T G G K A P R K Q	H3 1-19	R2me2a	K4me1	R8me2s	K9me1	free
161	G17	A Rme2a T Kme2 Q T A Rme2s Kme1 S T G G K A P R K Q	H3 1-19	R2me2a	K4me2	R8me2s	K9me1	free
162	G18	A Rme2a T Kme3 Q T A Rme2s Kme1 S T G G K A P R K Q	H3 1-19	R2me2a	K4me3	R8me2s	K9me1	free
163	G19	A Rme2a T Kac Q T A Rme2s Kme1 S T G G K A P R K Q	H3 1-19	R2me2a	K4ac	R8me2s	K9me1	free
164	G20	A Rme2s T Kme1 Q T A Rme2s Kme2 S T G G K A P R K Q	H3 1-19	R2me2s	K4me1	R8me2s	K9me2	free
165	G21	A Rme2s T Kme2 Q T A Rme2s Kme2 S T G G K A P R K Q	H3 1-19	R2me2s	K4me2	R8me2s	K9me2	free
166	G22	A Rme2s T Kme3 Q T A Rme2s Kme2 S T G G K A P R K Q	H3 1-19	R2me2s	K4me3	R8me2s	K9me2	free
167	G23	A Rme2s T Kac Q T A Rme2s Kme2 S T G G K A P R K Q	H3 1-19	R2me2s	K4ac	R8me2s	K9me2	free

168	G24	A Rme2a T Kme1 Q T A Rme2s Kme2 S T G G K A P R K Q	H3 1-19	R2me2a	K4me1	R8me2s	K9me2	free
169	H 1	A Rme2a T Kme2 Q T A Rme2s Kme2 S T G G K A P R K Q	H3 1-19	R2me2a	K4me2	R8me2s	K9me2	free
170	H 2	A Rme2a T Kme3 Q T A Rme2s Kme2 S T G G K A P R K Q	H3 1-19	R2me2a	K4me3	R8me2s	K9me2	free
171	H 3	A Rme2a T Kac Q T A Rme2s Kme2 S T G G K A P R K Q	H3 1-19	R2me2a	K4ac	R8me2s	K9me2	free
172	H 4	A Rme2s T Kme1 Q T A Rme2s Kme3 S T G G K A P R K Q	H3 1-19	R2me2s	K4me1	R8me2s	K9me3	free
173	H 5	A Rme2s T Kme2 Q T A Rme2s Kme3 S T G G K A P R K Q	H3 1-19	R2me2s	K4me2	R8me2s	K9me3	free
174	H 6	A Rme2s T Kme3 Q T A Rme2s Kme3 S T G G K A P R K Q	H3 1-19	R2me2s	K4me3	R8me2s	K9me3	free
175	H 7	A Rme2s T Kac Q T A Rme2s Kme3 S T G G K A P R K Q	H3 1-19	R2me2s	K4ac	R8me2s	K9me3	free
176	H 8	A Rme2a T Kme1 Q T A Rme2s Kme3 S T G G K A P R K Q	H3 1-19	R2me2a	K4me1	R8me2s	K9me3	free
177	H 9	A Rme2a T Kme2 Q T A Rme2s Kme3 S T G G K A P R K Q	H3 1-19	R2me2a	K4me2	R8me2s	K9me3	free
178	H10	A Rme2a T Kme3 Q T A Rme2s Kme3 S T G G K A P R K Q	H3 1-19	R2me2a	K4me3	R8me2s	K9me3	free
179	H11	A Rme2a T Kac Q T A Rme2s Kme3 S T G G K A P R K Q	H3 1-19	R2me2a	K4ac	R8me2s	K9me3	free
180	H12	A Rme2s T Kme1 Q T A Rme2s Kac S T G G K A P R K Q	H3 1-19	R2me2s	K4me1	R8me2s	K9ac	free
181	H13	A Rme2s T Kme2 Q T A Rme2s Kac S T G G K A P R K Q	H3 1-19	R2me2s	K4me2	R8me2s	K9ac	free
182	H14	A Rme2s T Kme3 Q T A Rme2s Kac S T G G K A P R K Q	H3 1-19	R2me2s	K4me3	R8me2s	K9ac	free
183	H15	A Rme2s T Kac Q T A Rme2s Kac S T G G K A P R K Q	H3 1-19	R2me2s	K4ac	R8me2s	K9ac	free
184	H16	A Rme2a T Kme1 Q T A Rme2s Kac S T G G K A P R K Q	H3 1-19	R2me2a	K4me1	R8me2s	K9ac	free
185	H17	A Rme2a T Kme2 Q T A Rme2s Kac S T G G K A P R K Q	H3 1-19	R2me2a	K4me2	R8me2s	K9ac	free
186	H18	A Rme2a T Kme3 Q T A Rme2s Kac S T G G K A P R K Q	H3 1-19	R2me2a	K4me3	R8me2s	K9ac	free
187	H19	A Rme2a T Kac Q T A Rme2s Kac S T G G K A P R K Q	H3 1-19	R2me2a	K4ac	R8me2s	K9ac	free
188	H20	A Rme2s T Kme1 Q T A Rme2a Kme1 S T G G K A P R K Q	H3 1-19	R2me2s	K4me1	R8me2a	K9me1	free
189	H21	A Rme2s T Kme2 Q T A Rme2a Kme1 S T G G K A P R K Q	H3 1-19	R2me2s	K4me2	R8me2a	K9me1	free
190	H22	A Rme2s T Kme3 Q T A Rme2a Kme1 S T G G K A P R K Q	H3 1-19	R2me2s	K4me3	R8me2a	K9me1	free
191	H23	A Rme2s T Kac Q T A Rme2a Kme1 S T G G K A P R K Q	H3 1-19	R2me2s	K4ac	R8me2a	K9me1	free
192	H24	A Rme2a T Kme1 Q T A Rme2a Kme1 S T G G K A P R K Q	H3 1-19	R2me2a	K4me1	R8me2a	K9me1	free
193	I 1	A Rme2a T Kme2 Q T A Rme2a Kme1 S T G G K A P R K Q	H3 1-19	R2me2a	K4me2	R8me2a	K9me1	free
194	I 2	A Rme2a T Kme3 Q T A Rme2a Kme1 S T G G K A P R K Q	H3 1-19	R2me2a	K4me3	R8me2a	K9me1	free
195	I 3	A Rme2a T Kac Q T A Rme2a Kme1 S T G G K A P R K Q	H3 1-19	R2me2a	K4ac	R8me2a	K9me1	free
196	I 4	A Rme2s T Kme1 Q T A Rme2a Kme2 S T G G K A P R K Q	H3 1-19	R2me2s	K4me1	R8me2a	K9me2	free
197	I 5	A Rme2s T Kme2 Q T A Rme2a Kme2 S T G G K A P R K Q	H3 1-19	R2me2s	K4me2	R8me2a	K9me2	free
198	I 6	A Rme2s T Kme3 Q T A Rme2a Kme2 S T G G K A P R K Q	H3 1-19	R2me2s	K4me3	R8me2a	K9me2	free
199	I 7	A Rme2s T Kac Q T A Rme2a Kme2 S T G G K A P R K Q	H3 1-19	R2me2s	K4ac	R8me2a	K9me2	free
200	I 8	A Rme2a T Kme1 Q T A Rme2a Kme2 S T G G K A P R K Q	H3 1-19	R2me2a	K4me1	R8me2a	K9me2	free
201	I 9	A Rme2a T Kme2 Q T A Rme2a Kme2 S T G G K A P R K Q	H3 1-19	R2me2a	K4me2	R8me2a	K9me2	free
202	I10	A Rme2a T Kme3 Q T A Rme2a Kme2 S T G G K A P R K Q	H3 1-19	R2me2a	K4me3	R8me2a	K9me2	free
203	I11	A Rme2a T Kac Q T A Rme2a Kme2 S T G G K A P R K Q	H3 1-19	R2me2a	K4ac	R8me2a	K9me2	free
204	I12	A Rme2s T Kme1 Q T A Rme2a Kme3 S T G G K A P R K Q	H3 1-19	R2me2s	K4me1	R8me2a	K9me3	free
205	I13	A Rme2s T Kme2 Q T A Rme2a Kme3 S T G G K A P R K Q	H3 1-19	R2me2s	K4me2	R8me2a	K9me3	free
206	I14	A Rme2s T Kme3 Q T A Rme2a Kme3 S T G G K A P R K Q	H3 1-19	R2me2s	K4me3	R8me2a	K9me3	free
207	I15	A Rme2s T Kac Q T A Rme2a Kme3 S T G G K A P R K Q	H3 1-19	R2me2s	K4ac	R8me2a	K9me3	free
208	I16	A Rme2a T Kme1 Q T A Rme2a Kme3 S T G G K A P R K Q	H3 1-19	R2me2a	K4me1	R8me2a	K9me3	free
209	I17	A Rme2a T Kme2 Q T A Rme2a Kme3 S T G G K A P R K Q	H3 1-19	R2me2a	K4me2	R8me2a	K9me3	free
210	I18	A Rme2a T Kme3 Q T A Rme2a Kme3 S T G G K A P R K Q	H3 1-19	R2me2a	K4me3	R8me2a	K9me3	free
211	I19	A Rme2a T Kac Q T A Rme2a Kme3 S T G G K A P R K Q	H3 1-19	R2me2a	K4ac	R8me2a	K9me3	free
212	I20	A Rme2s T Kme1 Q T A Rme2a Kac S T G G K A P R K Q	H3 1-19	R2me2s	K4me1	R8me2a	K9ac	free
213	I21	A Rme2s T Kme2 Q T A Rme2a Kac S T G G K A P R K Q	H3 1-19	R2me2s	K4me2	R8me2a	K9ac	free
214	I22	A Rme2s T Kme3 Q T A Rme2a Kac S T G G K A P R K Q	H3 1-19	R2me2s	K4me3	R8me2a	K9ac	free
215	I23	A Rme2s T Kac Q T A Rme2a Kac S T G G K A P R K Q	H3 1-19	R2me2s	K4ac	R8me2a	K9ac	free
216	I24	A Rme2a T Kme1 Q T A Rme2a Kac S T G G K A P R K Q	H3 1-19	R2me2a	K4me1	R8me2a	K9ac	free
217	J 1	A Rme2a T Kme2 Q T A Rme2a Kac S T G G K A P R K Q	H3 1-19	R2me2a	K4me2	R8me2a	K9ac	free
218	J 2	A Rme2a T Kme3 Q T A Rme2a Kac S T G G K A P R K Q	H3 1-19	R2me2a	K4me3	R8me2a	K9ac	free
219	J 3	A Rme2a T Kac Q T A Rme2a Kac S T G G K A P R K Q	H3 1-19	R2me2a	K4ac	R8me2a	K9ac	free
220	J 4	A R K S T G G K A P R K Q L A T K A A R	H3 7-26	unmod				acetylated
221	J 5	A R K S T G G Kac A P R K Q L A T K A A R	H3 7-26	K14ac				acetylated
222	J 6	A R K pS T G G Kac A P R K Q L A T K A A R	H3 7-26	K14ac	S10P			acetylated
223	J 7	A R K S pT G G Kac A P R K Q L A T K A A R	H3 7-26	K14ac	T11P			acetylated
224	J 8	A R K S T G G K A P Rme2s K Q L A T K A A R	H3 7-26	R17me2s				acetylated

225	J 9	A R K S T G G K A P Rme2a K Q L A T K A A R	H3 7-26	R17me2a				acetylated
226	J10	A R K S T G G K A P Cit K Q L A T K A A R	H3 7-26	R17Citr				acetylated
227	J11	A R K S T G G K A P R Kac Q L A T K A A R	H3 7-26	K18ac				acetylated
228	J12	A R K S T G G Kac A P Rme2s K Q L A T K A A R	H3 7-26	K14ac	R17me2s			acetylated
229	J13	A R K S T G G Kac A P Rme2a K Q L A T K A A R	H3 7-26	K14ac	R17me2a			acetylated
230	J14	A R K S T G G Kac A P R Kac Q L A T K A A R	H3 7-26	K14ac	K18ac			acetylated
231	J15	A R K S T G G K A P Rme2s Kac Q L A T K A A R	H3 7-26	R17me2s	K18ac			acetylated
232	J16	A R K S T G G K A P Rme2a Kac Q L A T K A A R	H3 7-26	R17me2a	K18ac			acetylated
233	J17	A R K S T G G K A P Cit Kac Q L A T K A A R	H3 7-26	R17Citr	K18ac			acetylated
234	J18	A R K S T G G Kac A P Rme2s Kac Q L A T K A A R	H3 7-26	K14ac	R17me2s	K18ac		acetylated
235	J19	A R K S T G G Kac A P Rme2a Kac Q L A T K A A R	H3 7-26	K14ac	R17me2a	K18ac		acetylated
236	J20	P R K Q L A T K A A R K S A P A T G G	H3 16-35	unmod				acetylated
237	J21	P R K Q L A T K A A Rme2s K S A P A T G G	H3 16-35	R26me2s				acetylated
238	J22	P R K Q L A T K A A Rme2a K S A P A T G G	H3 16-35	R26me2a				acetylated
239	J23	P R K Q L A T K A A Cit K S A P A T G G	H3 16-35	R26Citr				acetylated
240	J24	P R K Q L A T K A A R Kme1 S A P A T G G	H3 16-35	K27me1				acetylated
241	K 1	P R K Q L A T K A A R Kme2 S A P A T G G	H3 16-35	K27me2				acetylated
242	K 2	P R K Q L A T K A A R Kme3 S A P A T G G	H3 16-35	K27me3				acetylated
243	K 3	P R K Q L A T K A A R Kac S A P A T G G	H3 16-35	K27ac				acetylated
244	K 4	P R K Q L A T K A A R K pS A P A T G G	H3 16-35	S28P				acetylated
245	K 5	P R K Q L A T K A A Rme2s Kme1 S A P A T G G	H3 16-35	R26me2s	K27me1			acetylated
246	K 6	P R K Q L A T K A A Rme2s Kme2 S A P A T G G	H3 16-35	R26me2s	K27me2			acetylated
247	K 7	P R K Q L A T K A A Rme2s Kme3 S A P A T G G	H3 16-35	R26me2s	K27me3			acetylated
248	K 8	P R K Q L A T K A A Rme2s Kac S A P A T G G	H3 16-35	R26me2s	K27ac			acetylated
249	K 9	P R K Q L A T K A A Rme2s K pS A P A T G G	H3 16-35	R26me2s	S28P			acetylated
250	K10	P R K Q L A T K A A Rme2a Kme1 S A P A T G G	H3 16-35	R26me2a	K27me1			acetylated
251	K11	P R K Q L A T K A A Rme2a Kme2 S A P A T G G	H3 16-35	R26me2a	K27me2			acetylated
252	K12	P R K Q L A T K A A Rme2a Kme3 S A P A T G G	H3 16-35	R26me2a	K27me3			acetylated
253	K13	P R K Q L A T K A A Rme2a Kac S A P A T G G	H3 16-35	R26me2a	K27ac			acetylated
254	K14	P R K Q L A T K A A Rme2a K pS A P A T G G	H3 16-35	R26me2a	S28P			acetylated
255	K15	P R K Q L A T K A A Cit Kme1 S A P A T G G	H3 16-35	R26Citr	K27me1			acetylated
256	K16	P R K Q L A T K A A Cit Kme2 S A P A T G G	H3 16-35	R26Citr	K27me2			acetylated
257	K17	P R K Q L A T K A A Cit Kme3 S A P A T G G	H3 16-35	R26Citr	K27me3			acetylated
258	K18	P R K Q L A T K A A Cit K pS A P A T G G	H3 16-35	R26Citr	S28P			acetylated
259	K19	P R K Q L A T K A A R Kme1 pS A P A T G G	H3 16-35	K27me1	S28P			acetylated
260	K20	P R K Q L A T K A A R Kme2 pS A P A T G G	H3 16-35	K27me2	S28P			acetylated
261	K21	P R K Q L A T K A A R Kme3 pS A P A T G G	H3 16-35	K27me3	S28P			acetylated
262	K22	P R K Q L A T K A A R Kac pS A P A T G G	H3 16-35	K27ac	S28P			acetylated
263	K23	P R K Q L A T K A A Rme2s Kme1 pS A P A T G G	H3 16-35	R26me2s	K27me1	S28P		acetylated
264	K24	P R K Q L A T K A A Rme2s Kme2 pS A P A T G G	H3 16-35	R26me2s	K27me2	S28P		acetylated
265	L 1	P R K Q L A T K A A Rme2s Kme3 pS A P A T G G	H3 16-35	R26me2s	K27me3	S28P		acetylated
266	L 2	P R K Q L A T K A A Rme2s Kac pS A P A T G G	H3 16-35	R26me2s	K27ac	S28P		acetylated
267	L 3	P R K Q L A T K A A Rme2a Kme1 pS A P A T G G	H3 16-35	R26me2a	K27me1	S28P		acetylated
268	L 4	P R K Q L A T K A A Rme2a Kme2 pS A P A T G G	H3 16-35	R26me2a	K27me2	S28P		acetylated
269	L 5	P R K Q L A T K A A Rme2a Kme3 pS A P A T G G	H3 16-35	R26me2a	K27me3	S28P		acetylated
270	L 6	P R K Q L A T K A A Rme2a Kac pS A P A T G G	H3 16-35	R26me2a	K27ac	S28P		acetylated
271	L 7	R K S A P A T G G V K K P H R Y R P G	H3 26-45	unmod				acetylated
272	L 8	R K S A P A T G G V Kme1 K P H R Y R P G	H3 26-45	K36me1				acetylated
273	L 9	R K S A P A T G G V Kme2 K P H R Y R P G	H3 26-45	K36me2				acetylated
274	L10	R K S A P A T G G V Kme3 K P H R Y R P G	H3 26-45	K36me3				acetylated
275	L11	R K S A P A T G G V Kac K P H R Y R P G	H3 26-45	K36ac				acetylated
276	L12	S G R G K G G K G L G K G G A K R H R	H4 1-19	unmod				free
277	L13	pS G R G K G G K G L G K G G A K R H R	H4 1-19	S1P				free
278	L14	S G Rme2s G K G G K G L G K G G A K R H R	H4 1-19	R3me2s				free
279	L15	S G Rme2a G K G G K G L G K G G A K R H R	H4 1-19	R3me2a				free
280	L16	S G R G Kac G G K G L G K G G A K R H R	H4 1-19	K5ac				free
281	L17	S G R G K G G Kac G L G K G G A K R H R	H4 1-19	K8ac				free

282	L18	S G R G K G G K G L G Kac G G A K R H R	H4 1-19	K12ac				free
283	L19	S G R G K G G K G L G K G G A Kac R H R	H4 1-19	K16ac				free
284	L20	pS G Rme2s G K G G K G L G K G G A K R H R	H4 1-19	S1P	R3me2s			free
285	L21	pS G Rme2a G K G G K G L G K G G A K R H R	H4 1-19	S1P	R3me2a			free
286	L22	pS G R G Kac G G K G L G K G G A K R H R	H4 1-19	S1P	K5ac			free
287	L23	S G Rme2s G Kac G G K G L G K G G A K R H R	H4 1-19	R3me2s	K5ac			free
288	L24	S G Rme2s G K G G Kac G L G K G G A K R H R	H4 1-19	R3me2s	K8ac			free
289	M 1	S G Rme2a G Kac G G K G L G K G G A K R H R	H4 1-19	R3me2a	K5ac			free
290	M 2	S G Rme2a G K G G Kac G L G K G G A K R H R	H4 1-19	R3me2a	K8ac			free
291	M 3	S G R G Kac G G Kac G L G K G G A K R H R	H4 1-19	K5ac	K8ac			free
292	M 4	S G R G K G G Kac G L G Kac G G A K R H R	H4 1-19	K8ac	K12ac			free
293	M 5	S G R G K G G Kac G L G K G G A Kac R H R	H4 1-19	K8ac	K16ac			free
294	M 6	S G R G K G G K G L G Kac G G A Kac R H R	H4 1-19	K12ac	K16ac			free
295	M 7	pS G Rme2s G Kac G G K G L G K G G A K R H R	H4 1-19	S1P	R3me2s	K5ac		free
296	M 8	pS G Rme2a G Kac G G K G L G K G G A K R H R	H4 1-19	S1P	R3me2a	K5ac		free
297	M 9	S G Rme2s G Kac G G Kac G L G K G G A K R H R	H4 1-19	R3me2s	K5ac	K8ac		free
298	M10	S G Rme2a G Kac G G Kac G L G K G G A K R H R	H4 1-19	R3me2a	K5ac	K8ac		free
299	M11	S G R G Kac G G Kac G L G Kac G G A K R H R	H4 1-19	K5ac	K8ac	K12ac		free
300	M12	S G R G K G G Kac G L G Kac G G A Kac R H R	H4 1-19	K8ac	K12ac	K16ac		free
301	M13	pS G Rme2s G Kac G G Kac G L G K G G A K R H R	H4 1-19	S1P	R3me2s	K5ac	K8ac	free
302	M14	pS G Rme2a G Kac G G Kac G L G K G G A K R H R	H4 1-19	S1P	R3me2a	K5ac	K8ac	free
303	M15	S G Rme2s G Kac G G Kac G L G Kac G G A K R H R	H4 1-19	R3me2s	K5ac	K8ac	K12ac	free
304	M16	S G Rme2a G Kac G G Kac G L G Kac G G A K R H R	H4 1-19	R3me2a	K5ac	K8ac	K12ac	free
305	M17	S G R G Kac G G Kac G L G Kac G G A Kac R H R	H4 1-19	K5ac	K8ac	K12ac	K16ac	free
306	M18	G K G G A K R H R K V L R D N I Q G I T	H4 11-30	unmod				acetylated
307	M19	G Kac G G A K R H R K V L R D N I Q G I T	H4 11-30	K12ac				acetylated
308	M20	G K G G A Kac R H R K V L R D N I Q G I T	H4 11-30	K16ac				acetylated
309	M21	G K G G A K Rme2s H R K V L R D N I Q G I T	H4 11-30	R17me2s				acetylated
310	M22	G K G G A K Rme2a H R K V L R D N I Q G I T	H4 11-30	R17me2a				acetylated
311	M23	G K G G A K R H Rme2s K V L R D N I Q G I T	H4 11-30	R19me2s				acetylated
312	M24	G K G G A K R H Rme2a K V L R D N I Q G I T	H4 11-30	R19me2a				acetylated
313	N 1	G K G G A K R H R Kme1 V L R D N I Q G I T	H4 11-30	K20me1				acetylated
314	N 2	G K G G A K R H R Kme2 V L R D N I Q G I T	H4 11-30	K20me2				acetylated
315	N 3	G K G G A K R H R Kme3 V L R D N I Q G I T	H4 11-30	K20me3				acetylated
316	N 4	G K G G A K R H R Kac V L R D N I Q G I T	H4 11-30	K20ac				acetylated
317	N 5	G K G G A K R H R K V L Rme2a D N I Q G I T	H4 11-30	R24me2a				acetylated
318	N 6	G K G G A K R H R K V L Rme2s D N I Q G I T	H4 11-30	R24me2s				acetylated
319	N 7	G Kac G G A Kac R H R K V L R D N I Q G I T	H4 11-30	K12ac	K16ac			acetylated
320	N 8	G K G G A Kac Rme2s H R K V L R D N I Q G I T	H4 11-30	K16ac	R17me2s			acetylated
321	N 9	G K G G A Kac Rme2a H R K V L R D N I Q G I T	H4 11-30	K16ac	R17me2a			acetylated
322	N10	G K G G A Kac R H Rme2s K V L R D N I Q G I T	H4 11-30	K16ac	R19me2s			acetylated
323	N11	G K G G A Kac R H Rme2a K V L R D N I Q G I T	H4 11-30	K16ac	R19me2a			acetylated
324	N12	G K G G A Kac R H R Kme1 V L R D N I Q G I T	H4 11-30	K16ac	K20me1			acetylated
325	N13	G K G G A Kac R H R Kme2 V L R D N I Q G I T	H4 11-30	K16ac	K20me2			acetylated
326	N14	G K G G A Kac R H R Kme3 V L R D N I Q G I T	H4 11-30	K16ac	K20me3			acetylated
327	N15	G K G G A Kac R H R Kac V L R D N I Q G I T	H4 11-30	K16ac	K20ac			acetylated
328	N16	G Kac G G A Kac R H R Kme1 V L R D N I Q G I T	H4 11-30	K12ac	K16ac	K20me1		acetylated
329	N17	G Kac G G A Kac R H R Kme2 V L R D N I Q G I T	H4 11-30	K12ac	K16ac	K20me2		acetylated
330	N18	G Kac G G A Kac R H R Kme3 V L R D N I Q G I T	H4 11-30	K12ac	K16ac	K20me3		acetylated
331	N19	G Kac G G A Kac R H R Kac V L R D N I Q G I T	H4 11-30	K12ac	K16ac	K20ac		acetylated
332	N20	G K G G A K R H Rme2a Kme1 V L R D N I Q G I T	H4 11-30	R19me2a	K20me1			acetylated
333	N21	G K G G A K R H Rme2a Kme2 V L R D N I Q G I T	H4 11-30	R19me2a	K20me2			acetylated
334	N22	G K G G A K R H Rme2a Kme3 V L R D N I Q G I T	H4 11-30	R19me2a	K20me3			acetylated
335	N23	G K G G A K R H Rme2a Kac V L R D N I Q G I T	H4 11-30	R19me2a	K20ac			acetylated
336	N24	G K G G A K R H Rme2s Kme1 V L R D N I Q G I T	H4 11-30	R19me2s	K20me1			acetylated
337	O 1	G K G G A K R H Rme2s Kme2 V L R D N I Q G I T	H4 11-30	R19me2s	K20me2			acetylated
338	O 2	G K G G A K R H Rme2s Kme3 V L R D N I Q G I T	H4 11-30	R19me2s	K20me3			acetylated

339	O 3	G K G G A K R H Rme2s Kac V L R D N I Q G I T	H4 11-30	R19me2s	K20ac				acetylated
340	O 4	G K G G A K R H R Kme1 V L Rme2a D N I Q G I T	H4 11-30	R24me2a	K20me1				acetylated
341	O 5	G K G G A K R H R Kme2 V L Rme2a D N I Q G I T	H4 11-30	R24me2a	K20me2				acetylated
342	O 6	G K G G A K R H R Kme3 V L Rme2a D N I Q G I T	H4 11-30	R24me2a	K20me3				acetylated
343	O 7	G K G G A K R H R Kac V L Rme2a D N I Q G I T	H4 11-30	R24me2a	K20ac				acetylated
344	O 8	G K G G A K R H R Kme1 V L Rme2s D N I Q G I T	H4 11-30	R24me2s	K20me1				acetylated
345	O 9	G K G G A K R H R Kme2 V L Rme2s D N I Q G I T	H4 11-30	R24me2s	K20me2				acetylated
346	O10	G K G G A K R H R Kme3 V L Rme2s D N I Q G I T	H4 11-30	R24me2s	K20me3				acetylated
347	O11	G K G G A K R H R Kac V L Rme2s D N I Q G I T	H4 11-30	R24me2s	K20ac				acetylated
348	O12	S G R G K Q G G K A R A K A K S R S S	H2a 1-19	unmod					free
349	O13	pS G R G K Q G G K A R A K A K S R S S	H2a 1-19	S1P					free
350	O14	S G R G Kac Q G G K A R A K A K S R S S	H2a 1-19	K5ac					free
351	O15	S G R G K Q G G Kac A R A K A K S R S S	H2a 1-19	K9ac					free
352	O16	S G R G K Q G G K A R A Kac A K S R S S	H2a 1-19	K13ac					free
353	O17	pS G R G Kac Q G G K A R A K A K S R S S	H2a 1-19	S1P	K5ac				free
354	O18	pS G R G K Q G G Kac A R A K A K S R S S	H2a 1-19	S1P	K9ac				free
355	O19	pS G R G K Q G G K A R A Kac A K S R S S	H2a 1-19	S1P	K13ac				free
356	O20	S G R G Kac Q G G Kac A R A K A K S R S S	H2a 1-19	K5ac	K9ac				free
357	O21	S G R G Kac Q G G K A R A Kac A K S R S S	H2a 1-19	K5ac	K13ac				free
358	O22	S G R G K Q G G Kac A R A Kac A K S R S S	H2a 1-19	K9ac	K13ac				free
359	O23	pS G R G Kac Q G G Kac A R A K A K S R S S	H2a 1-19	S1P	K5ac	K9ac			free
360	O24	pS G R G Kac Q G G K A R A Kac A K S R S S	H2a 1-19	S1P	K5ac	K13ac			free
361	P 1	pS G R G K Q G G Kac A R A Kac A K S R S S	H2a 1-19	S1P	K9ac	K13ac			free
362	P 2	S G R G Kac Q G G Kac A R A Kac A K S R S S	H2a 1-19	K5ac	K9ac	K13ac			free
363	P 3	pS G R G Kac Q G G Kac A R A Kac A K S R S S	H2a 1-19	S1P	K5ac	K9ac	K13ac		free
364	P 4	P D P A K S A P A P K K G S K K A V T	H2B 1-19	unmod					free
365	P 5	P D P A Kac S A P A P K K G S K K A V T	H2B 1-19	K5ac					free
366	P 6	P D P A K S A P A P K Kac G S K K A V T	H2B 1-19	K12ac					free
367	P 7	P D P A K S A P A P K K G pS K K A V T	H2B 1-19	S14P					free
368	P 8	P D P A K S A P A P K K G S Kac K A V T	H2B 1-19	K15ac					free
369	P 9	P D P A Kac S A P A P K Kac G S K K A V T	H2B 1-19	K5ac	K12ac				free
370	P10	P D P A Kac S A P A P K K G pS K K A V T	H2B 1-19	K5ac	S14P				free
371	P11	P D P A Kac S A P A P K K G S Kac K A V T	H2B 1-19	K5ac	K15ac				free
372	P12	P D P A K S A P A P K Kac G pS K K A V T	H2B 1-19	K12ac	S14P				free
373	P13	P D P A K S A P A P K Kac G S Kac K A V T	H2B 1-19	K12ac	K15ac				free
374	P14	P D P A K S A P A P K K G pS Kac K A V T	H2B 1-19	S14P	K15ac				free
375	P15	P D P A Kac S A P A P K Kac G pS K K A V T	H2B 1-19	K5ac	K12ac	S14P			free
376	P16	P D P A Kac S A P A P K Kac G S Kac K A V T	H2B 1-19	K5ac	K12ac	K15ac			free
377	P17	P D P A Kac S A P A P K K G pS Kac K A V T	H2B 1-19	K5ac	S14P	K15ac			free
378	P18	P D P A K S A P A P K Kac G pS Kac K A V T	H2B 1-19	K12ac	S14P	K15ac			free
379	P19	P D P A Kac S A P A P K Kac G pS Kac K A V T	H2B 1-19	K5ac	K12ac	S14P	K15ac		free
380	P20	Bio A A N W S H P Q F E K A A	Biotin, control peptide						biotinylated
381	P21	E Q K L I S E E D L A	c-myc tag						free
382	P22	HAc	neg. contol						acetylated
383	P23	K Kme1 Kme2 Kme3 Kac R Rme2s R Rme2a R Cit K Kme1 Kac Kme3 R K	background 01						acetylated
384	P24	R Rme2s K Kme1 Kac R Rme2a Kme2 K Kme3 R Kme1 Rme2s K Kac R K	background 02						acetylated

*CelluSpots™ arrays are manufactured under license by INTAVIS Bioanalytical Instruments AG.

REFERENCES

1 Casimiro MC, Crosariol M, Loro E, Ertel A, Yu Z, Dampier W *et al* (2012). ChIP sequencing of cyclin D1 reveals a transcriptional role in chromosomal instability in mice. *J Clin Invest* **122:** 833-843.

2 Madabhushi R, Gao F, Pfenning AR, Pan L, Yamakawa S, Seo J *et al* (2015). Activity-Induced DNA Breaks Govern the Expression of Neuronal Early-Response Genes. *Cell* **161:** 1592-1605.

3 Sahara S, Aoto M, Eguchi Y, Imamoto N, Yoneda Y, Tsujimoto Y (1999). Acinus is a caspase-3-activated protein required for apoptotic chromatin condensation. *Nature* **401:** 168-173.

4 Li C, Fan Y, Lan TH, Lambert NA, Wu G (2012). Rab26 modulates the cell surface transport of alpha2-adrenergic receptors from the Golgi. *J Biol Chem* **287:** 42784-42794.

5 Chen S, Jiao L, Shubbar M, Yang X, Liu X (2018). Unique Structural Platforms of Suz12 Dictate Distinct Classes of PRC2 for Chromatin Binding. *Mol Cell* **69:** 840-852 e845.

6 Graser S, Stierhof YD, Lavoie SB, Gassner OS, Lamla S, Le Clech M *et al* (2007). Cep164, a novel centriole appendage protein required for primary cilium formation. *J Cell Biol* **179:** 321-330.

7 Obri A, Ouararhni K, Papin C, Diebold ML, Padmanabhan K, Marek M *et al* (2014). ANP32E is a histone chaperone that removes H2A.Z from chromatin. *Nature* **505:** 648-653.

8 Fleischer TC, Yun UJ, Ayer DE (2003). Identification and characterization of three new components of the mSin3A corepressor complex. *Mol Cell Biol* **23:** 3456-3467.

9 Valle-Garcia D, Qadeer ZA, McHugh DS, Ghiraldini FG, Chowdhury AH, Hasson D *et al* (2016). ATRX binds to atypical chromatin domains at the 3' exons of zinc finger genes to preserve H3K9me3 enrichment. *Epigenetics* **11:** 398-414.

10 Bozhenok L, Wade PA, Varga-Weisz P (2002). WSTF-ISWI chromatin remodeling complex targets heterochromatic replication foci. *EMBO J* **21:** 2231-2241.

11 Liu N, Hargreaves VV, Zhu Q, Kurland JV, Hong J, Kim W *et al* (2018). Direct Promoter Repression by BCL11A Controls the Fetal to Adult Hemoglobin Switch. *Cell* **173:** 430-442 e417.

12 Cismasiu VB, Ghanta S, Duque J, Albu DI, Chen HM, Kasturi R *et al* (2006). BCL11B participates in the activation of IL2 gene expression in CD4+ T lymphocytes. *Blood* **108:** 2695-2702.

13 Pagan JK, Arnold J, Hanchard KJ, Kumar R, Bruno T, Jones MJ *et al* (2007). A novel corepressor, BCoR-L1, represses transcription through an interaction with CtBP. *J Biol Chem* **282:** 15248-15257.

14 Liu WD, Wang HW, Muguira M, Breslin MB, Lan MS (2006). INSM1 functions as a transcriptional repressor of the neuroD/beta2 gene through the recruitment of cyclin D1 and histone deacetylases. *Biochem J* **397:** 169-177.

15 Hu D, Mayeda A, Trembley JH, Lahti JM, Kidd VJ (2003). CDK11 complexes promote pre-mRNA splicing. *J Biol Chem* **278:** 8623-8629.

16 Bunnell BA, Heath LS, Adams DE, Lahti JM, Kidd VJ (1990). Increased expression of a 58-kDa protein kinase leads to changes in the CHO cell cycle. *Proc Natl Acad Sci U S A* **87:** 7467-7471.

17 Tanaka Y, Nureki O, Kurumizaka H, Fukai S, Kawaguchi S, Ikuta M *et al* (2001). Crystal structure of the CENP-B protein-DNA complex: the DNA-binding domains of CENP-B induce kinks in the CENP-B box DNA. *EMBO J* **20:** 6612-6618.

18 Tong JK, Hassig CA, Schnitzler GR, Kingston RE, Schreiber SL (1998). Chromatin deacetylation by an ATP-dependent nucleosome remodelling complex. *Nature* **395:** 917-921.

19 Sar F, Lindsey-Boltz LA, Subramanian D, Croteau DL, Hutsell SQ, Griffith JD *et al* (2004). Human claspin is a ring-shaped DNA-binding protein with high affinity to branched DNA structures. *J Biol Chem* **279:** 39289-39295.

20 Li Z, Pei XH, Yan J, Yan F, Cappell KM, Whitehurst AW *et al* (2014). CUL9 mediates the functions of the 3M complex and ubiquitylates survivin to maintain genome integrity. *Mol Cell* **54:** 805-819.

21 Hollenbach AD, McPherson CJ, Mientjes EJ, Iyengar R, Grosveld G (2002). Daxx and histone deacetylase II associate with chromatin through an interaction with core histones and the chromatin-associated protein Dek. *J Cell Sci* **115:** 3319-3330.

22 Tachibana M, Sugimoto K, Fukushima T, Shinkai Y (2001). Set domain-containing protein, G9a, is a novel lysine-preferring mammalian histone methyltransferase with hyperactivity and specific selectivity to lysines 9 and 27 of histone H3. *J Biol Chem* **276:** 25309-25317.

23 Lee AS, Kranzusch PJ, Doudna JA, Cate JH (2016). eIF3d is an mRNA cap-binding protein that is required for specialized translation initiation. *Nature* **536:** 96-99.

24 Morello LG, Coltri PP, Quaresma AJ, Simabuco FM, Silva TC, Singh G *et al* (2011). The human nucleolar protein FTSJ3 associates with NIP7 and functions in pre-rRNA processing. *PLoS One* **6:** e29174.

25 Morinaga T, Enomoto A, Shimono Y, Hirose F, Fukuda N, Dambara A *et al* (2005). GDNF-inducible zinc finger protein 1 is a sequence-specific transcriptional repressor that binds to the HOXA10 gene regulatory region. *Nucleic Acids Res* **33:** 4191-4201.

26 Fackelmayer FO, Dahm K, Renz A, Ramsperger U, Richter A (1994). Nucleic-acid-binding properties of hnRNP-U/SAF-A, a nuclear-matrix protein which binds DNA and RNA in vivo and in vitro. *Eur J Biochem* **221:** 749-757.

27 De I, Bessonov S, Hofele R, dos Santos K, Will CL, Urlaub H *et al* (2015). The RNA helicase Aquarius exhibits structural adaptations mediating its recruitment to spliceosomes. *Nat Struct Mol Biol* **22:** 138-144.

28 Ding XF, Luo C, Ren KQ, Zhang J, Zhou JL, Hu X *et al* (2008). Characterization and expression of a human KCTD1 gene containing the BTB domain, which mediates transcriptional repression and homomeric interactions. *DNA Cell Biol* **27:** 257-265.

29 Frescas D, Guardavaccaro D, Kuchay SM, Kato H, Poleshko A, Basrur V *et al* (2008). KDM2A represses transcription of centromeric satellite repeats and maintains the heterochromatic state. *Cell Cycle* **7:** 3539-3547.

30 Ding Z, Gillespie LL, Paterno GD (2003). Human MI-ER1 alpha and beta function as transcriptional repressors by recruitment of histone deacetylase 1 to their conserved ELM2 domain. *Mol Cell Biol* **23:** 250-258.

31 Nielsen JA, Berndt JA, Hudson LD, Armstrong RC (2004). Myelin transcription factor 1 (Myt1) modulates the proliferation and differentiation of oligodendrocyte lineage cells. *Mol Cell Neurosci* **25:** 111-123.

32 Parada CA, Roeder RG (1999). A novel RNA polymerase II-containing complex potentiates Tat-enhanced HIV-1 transcription. *EMBO J* **18:** 3688-3701.

33 Wang X, Takenaka K, Takeda S (2010). PTIP promotes DNA double-strand break repair through homologous recombination. *Genes Cells* **15:** 243-254.

34 Lee MG, Norman J, Shilatifard A, Shiekhattar R (2007). Physical and functional association of a trimethyl H3K4 demethylase and Ring6a/MBLR, a polycomb-like protein. *Cell* **128:** 877-887.

35 Nair SS, Mishra SK, Yang Z, Balasenthil S, Kumar R, Vadlamudi RK (2004). Potential role of a novel transcriptional coactivator PELP1 in histone H1 displacement in cancer cells. *Cancer Res* **64:** 6416-6423.

36 Gao SS, Guan H, Yan S, Hu S, Song M, Guo ZP *et al* (2020). TIP60 K430 SUMOylation attenuates its interaction with DNA-PKcs in S-phase cells: Facilitating homologous recombination and emerging target for cancer therapy. *Sci Adv* **6:** eaba7822.

37 Escoffier J, Lee HC, Yassine S, Zouari R, Martinez G, Karaouzene T *et al* (2016). Homozygous mutation of PLCZ1 leads to defective human oocyte activation and infertility that is not rescued by the WW-binding protein PAWP. *Hum Mol Genet* **25:** 878-891.

38 Kim KC, Geng L, Huang S (2003). Inactivation of a histone methyltransferase by mutations in human cancers. *Cancer Res* **63:** 7619-7623.

39 Gorlich D, Pante N, Kutay U, Aebi U, Bischoff FR (1996). Identification of different roles for RanGDP and RanGTP in nuclear protein import. *EMBO J* **15:** 5584-5594.

40 Shinsky SA, Monteith KE, Viggiano S, Cosgrove MS (2015). Biochemical reconstitution and phylogenetic comparison of human SET1 family core complexes involved in histone methylation. *J Biol Chem* **290:** 6361-6375.

41 Euskirchen G, Auerbach RK, Snyder M (2012). SWI/SNF chromatin-remodeling factors: multiscale analyses and diverse functions. *J Biol Chem* **287:** 30897-30905.

42 Ivanov D, Kwak YT, Guo J, Gaynor RB (2000). Domains in the SPT5 protein that modulate its transcriptional regulatory properties. *Mol Cell Biol* **20:** 2970-2983.

43 Raman M, Earnest S, Zhang K, Zhao Y, Cobb MH (2007). TAO kinases mediate activation of p38 in response to DNA damage. *EMBO J* **26:** 2005-2014.

44 Engelen E, Janssens RC, Yagita K, Smits VA, van der Horst GT, Tamanini F (2013). Mammalian TIMELESS is involved in period determination and DNA damage-dependent phase advancing of the circadian clock. *PLoS One* **8:** e56623.

45 Reuter S, Bartelmann M, Vogt M, Geisen C, Napierski I, Kahn T *et al* (1998). APM-1, a novel human gene, identified by aberrant co-transcription with papillomavirus oncogenes in a cervical carcinoma cell line, encodes a BTB/POZ-zinc finger protein with growth inhibitory activity. *EMBO J* **17:** 215-222.

46 Sanchez-Tillo E, Lazaro A, Torrent R, Cuatrecasas M, Vaquero EC, Castells A *et al* (2010). ZEB1 represses E-cadherin and induces an EMT by recruiting the SWI/SNF chromatin-remodeling protein BRG1. *Oncogene* **29:** 3490-3500.

47 Sakata N, Kaneko S, Ikeno S, Miura Y, Nakabayashi H, Dong XY *et al* (2014). TGF- beta Signaling Cooperates with AT Motif-Binding Factor-1 for Repression of the alpha -Fetoprotein Promoter. *J Signal Transduct* **2014:** 970346.
